# The NagY regulator: A member of the BglG/SacY antiterminator family conserved in *Enterococcus faecalis* and involved in virulence

**DOI:** 10.3389/fmicb.2022.1070116

**Published:** 2023-02-17

**Authors:** Diane Soussan, Marine Salze, Pierre Ledormand, Nicolas Sauvageot, Amine Boukerb, Olivier Lesouhaitier, Gwennaele Fichant, Alain Rincé, Yves Quentin, Cécile Muller

**Affiliations:** ^1^Unité de Recherche Communication Bactérienne et Stratégies Anti-infectieuses, CBSA UR4312, Normandie Université, UNICAEN, Caen, France; ^2^Fédération de Recherche SeSAD, Normandie Université, UNICAEN, Caen, France; ^3^Plateforme de Génomique, CBSA EA4312, Normandie Université, UNIROUEN, Évreux, France; ^4^Laboratoire de Microbiologie et Génétique Moléculaires, UMR5100, Centre de Biologie Intégrative (CBI), Université de Toulouse, CNRS, Université Paul Sabatier, Toulouse, France

**Keywords:** antiterminator, regulation, N-acetylglucosamine, glycosaminoglycans, virulence, comparative genomics, phylogenetic, *Enterococcus faecalis*

## Abstract

*Enterococcus faecalis* is a commensal bacterium of the gastrointestinal tract but also a major nosocomial pathogen. This bacterium uses regulators like BglG/SacY family of transcriptional antiterminators to adapt its metabolism during host colonization. In this report, we investigated the role of the BglG/SacY family antiterminator NagY in the regulation of the *nagY-nagE* operon in presence of N-acetylglucosamine, with *nagE* encoding a transporter of this carbohydrate, as well as the expression of the virulence factor HylA. We showed that this last protein is involved in biofilm formation and glycosaminoglycans degradation that are important features in bacterial infection, confirmed in the *Galleria mellonella* model. In order to elucidate the evolution of these actors, we performed phylogenomic analyses on *E*. *faecalis* and *Enterococcaceae* genomes, identified orthologous sequences of NagY, NagE, and HylA, and we report their taxonomic distribution. The study of the conservation of the upstream region of *nagY* and *hylA* genes showed that the molecular mechanism of NagY regulation involves ribonucleic antiterminator sequence overlapping a rho-independent terminator, suggesting a regulation conforming to the canonical model of BglG/SacY family antiterminators. In the perspective of opportunism understanding, we offer new insights into the mechanism of host sensing thanks to the NagY antiterminator and its targets expression.

## 1. Introduction

According to the Genome Taxonomy Database Enterococci are composed of 13 genera (*Enterococcus*, *Enterococcus*-A to J, *Melissococcus*, and *Tetragenococcus*). They belong to the *Enteroccocaceae* family and to the order *Lactobacillales*, with other families of medical importance such as *Streptococcaceae* (Ludwig et al., 2009; García-Solache and Rice, 2019). Enterococci are Gram-positive facultative anaerobic bacteria, commonly found in mammal’s intestinal microbiota, and also major health care-associated infection pathogens, especially *Enterococcus faecalis* and *Enterococcus faecium* (Hendrickx et al., 2009; Fiore et al., 2019). As well-documented pathogens, enterococci are associated with various clinical manifestations including urinary tract infections, bacteremia, or endocarditis and they can also be recovered from cultures of intra-abdominal, pelvic, and soft tissue infections (Agudelo Higuita and Huycke, 2014). *Enterococcus faecalis* is reported to be responsible for 10% of all infective endocarditis cases (Fernández-Hidalgo et al., 2020; Barnes et al., 2021), and *Enterococcus* spp. is considered as the third causative agent of these infections in Europe (Habib et al., 2019).

*Enterococcus faecalis* and *E*. *faecium* present numerous intrinsic and acquired resistances to antibiotics, that makes treatment of enterococcal infections particularly challenging (Agudelo Higuita and Huycke, 2014; Faron et al., 2016). Indeed, enterococci are intrinsically resistant to β-lactams, aminoglycosides, or lincosamides, and they can acquire resistance to antibiotics of all classes that have so far been introduced for therapy, like lipopeptides, cyclines, or glycopeptides (Fiore et al., 2019). These characteristics, which distinguish them from their ancestors, allow them to persist in the modern hospital environment (Lebreton et al., 2017). At the beginning of the 21st Century, the rapid increase of vancomycin resistance in enterococci raised alarms because this antibiotic was formerly designated as “last resort” for the treatment of Gram-positive multidrug-resistant bacteria (Faron et al., 2016; Fiore et al., 2019).

The *Enterococcus* spp. transition from commensal to pathogen is observed as a result of overgrowth in the colon, which increases the risk by simple numerical probability of dissemination into the bloodstream and in other sites, especially in susceptible hosts (Fiore et al., 2019; Kao and Kline, 2019). The ability to obtain nutrients within the competitive environment of the gut is also an important aspect of colonization efficiency (Ramsey et al., 2014; Fiore et al., 2019). The *E*. *faecalis* metabolism undergoes significant expression changes even more important than those observed for virulence factor genes during an infection of mouse peritoneum (Muller et al., 2015). Transcriptomic studies also showed that resistance abilities during mice infection or when cells are exposed to stress during colonization are more dependent on metabolism or stress response genes than virulence traits (Muller et al., 2015; Salze et al., 2020a). In this context, enzyme like hyaluronidase was poorly investigated in enterococci, even though most Gram-positive pathogenic bacteria produce these proteins in their survival and infection strategies. The hyaluronidases are capable of cutting β-1,4 glycosidic bonds between the N-acetyl-glucosamine (NAG) and the D-glucuronic acid that composed the hyaluronic acid (HA; [-D-glucuronic acid-β1,3-N-acetyl-D-glucosamine-β1,4-]n; Stern and Jedrzejas, 2006). HA is the most widespread glycosaminoglycans (GAGs) with chondroitin and heparin, which are found as components of the extracellular matrix (ECM; Theocharis et al., 2016). As a monosaccharide capable of β-binding to another monosaccharide, the NAG is considered as a β-glucoside. Therefore, the degradation of HA by hyaluronidases can provide two advantages: firstly, they can facilitate bacterial spread by degradation of HA composing the host ECM, and secondly, they can provide a source for their carbon and energy requirements (Stern and Jedrzejas, 2006; Kawai et al., 2018).

**Figure 1:**
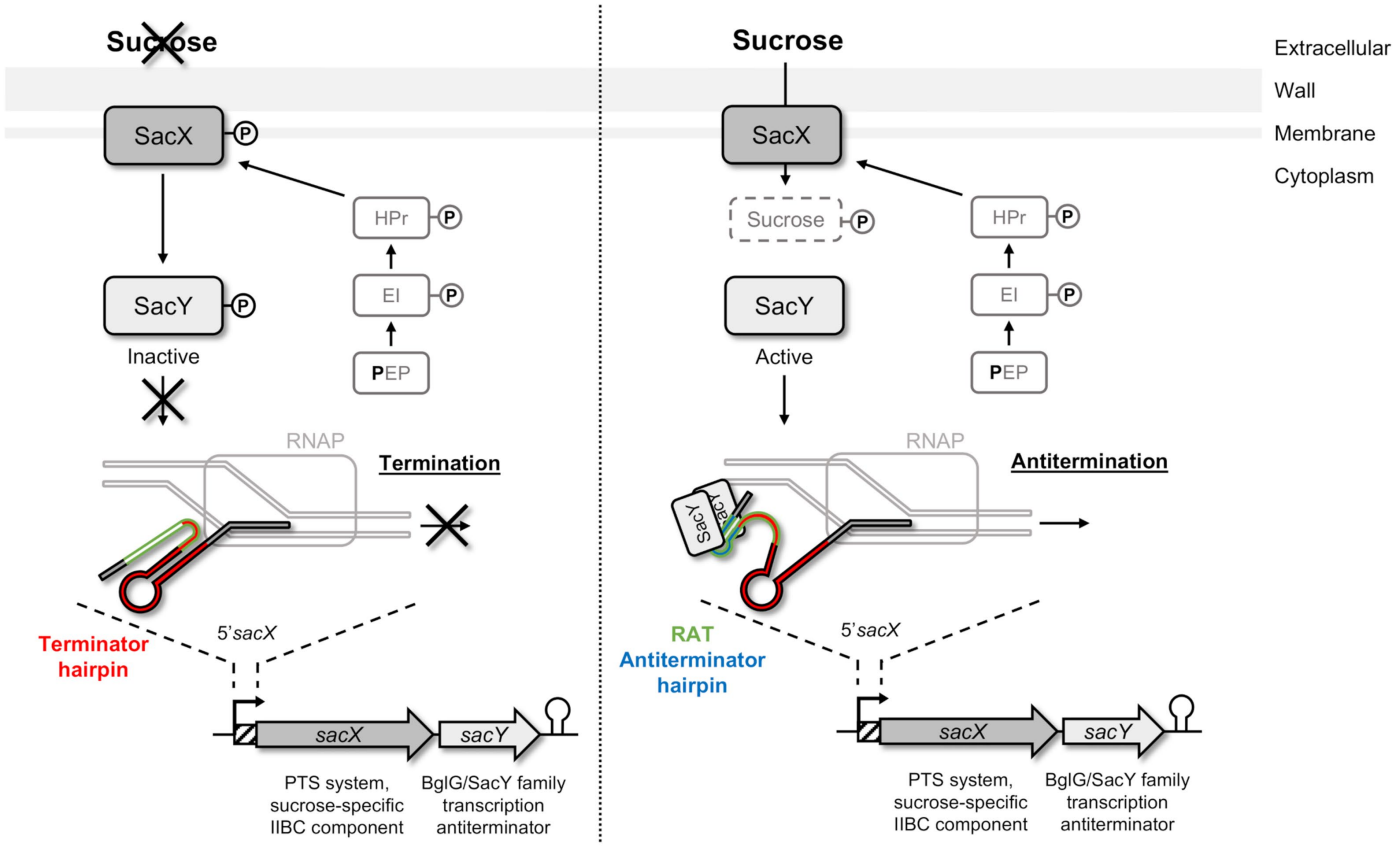
Schematic representation of SacY mechanism in *Bacillus subtilis*. SacX EIIBC enzyme is phosphorylated by HPr from the phosphotransferase system (PTS) pathway, and regulation of *sacX-sacY* operon occurs depending on the presence of sucrose. In the absence of the specific carbohydrate (left pannel), SacY is phosphorylated and inactivated by SacX. The *sacX-sacY* transcription is initiated by RNA polymerase (RNAP) but stops by the closing of the terminator hairpin (indicated in red). In the presence of this carbohydrate (right panel), SacY binds its own mRNA on the untranslated region 5’*sacX*, and more specifically on the ribonucleic antiterminator sequence (RAT; indicated in green). This interaction promotes the antitermination hairpin (indicated in blue), which prevents the closure of the terminator hairpin, and makes the transcription terminator present on 5’*sacX* ineffective (anti-termination). Sucrose is then phosphorylated by SacX during its import into the cell and metabolized (Clerte et al., 2013).

To metabolize the nutrients, the bacteria must first internalize them by different systems, especially the phosphoenolpyruvate-sugar phosphotransferase system (PTS) involved in carbohydrates uptake by using the energy derived from glycolysis-produced phosphoenolpyruvate (PEP; Kundig et al., 1964; Deutscher et al., 2006; Galinier and Deutscher, 2017). These systems are usually composed of several proteins: enzyme I (EI), heat-stable protein (HPr), and enzyme II (EII) that are activated successively by phosphorylation (Kundig et al., 1964; Deutscher et al., 2006). The EII is composed of the EIIA, EIIB, and EIIC (occasionally EIID) subunits, which can be combined and are specific of one substrate or small group of closely related carbohydrates. HPr is also involved in other regulatory mechanisms such as the carbon catabolite repression (CCR) for the orderly utilization of secondary carbon sources, or in the activity control of proteins containing PTS regulatory domains (PRDs), such as BglG/SacY family antiterminators (Prasad and Schaefler, 1974; Stülke, 2002; Görke, 2003; Deutscher et al., 2006; Görke and Stülke, 2008). Although the Bgl system was firstly described in *Escherichia coli*, such systems are highly conserved in bacteria and are also present in Gram-positive bacteria, like *Bacillus subtilis* with SacY involved in sucrose utilization (Steinmetz et al., 1988; Arnaud et al., 1996; Stülke et al., 1998; Tortosa et al., 2001). In *B*. *subtilis*, SacY is encoded in an operon with the *sacX* gene (Tortosa and Le Coq, 1995; Deutscher et al., 2006). SacX protein is a sucrose specific EIIBC, which has a role in SacY activation in absence of the carbohydrate (Tortosa et al., 2001). In the presence of sucrose, SacY binds to a specific and conserved sequence called ribonucleic antiterminator (RAT) located in the RNA 5′untranslated region (5′UTR) of the *sacXY* operon (Figure 1; Aymerich and Steinmetz, 1992; Tortosa et al., 2001; Clerte et al., 2013). This binding can lead to the opening of the terminator hairpin and therefore make the transcription terminator ineffective and allows transcription of the specific genes that are not usually transcribed (Aymerich and Steinmetz, 1992; Tortosa et al., 2001; Clerte et al., 2013).

Herein, we investigate the role of a BglG/SacY antiterminator homolog as a link between the metabolism and the opportunistic features of *E*. *faecalis*. An interesting aspect is that the β-glucosides metabolism was shown to be induced during infection (Muller et al., 2015), so we studied the regulation of this metabolism in *E*. *faecalis* by the *ef1515-ef1516* operon encoding a BglG/SacY-like antiterminator (NagY) and a NAG PTS transporter (NagE; Keffeler et al., 2021b). We analyzed the autoregulation mechanism of NagY, and its action on the expression of a hyaluronidase HylA, identified as a MSCRAMM (Microbial Surface Components Recognizing Adhesive Matrix Molecules). A phylogenomic approach was also used to complete this study in order to elucidate the processes at work in the evolution of the *nagY*, *nagE*, and *hylA* genes among a set of representative *Enterococcaceae* species and a large sample of *E*. *faecalis* strains. This comparative genomics approach allowed us to identify conserved RAT-like motifs involved in the regulation of the expression of these genes.

## 2. Materials and methods

### 2.1. Bacterial strains and growth conditions

The reference strain used in this study is *E*. *faecalis* V19, which corresponds to a plasmid-cured strain derived from the V583 strain of clinical origin (Paulsen et al., 2003). Overnight cultures were achieved in M17 medium supplemented with 0.5% glucose (GM17). *Escherichia coli* strains TOP10 (ThermoFisher, Waltham, MA, United States), NEB-5α (New England BioLabs, Ipswich, MA, United States), and M15 pRep4 (Qiagen, Hilden, Germany) were used for RNA *in vitro* production, mutant constructions, and recombinant protein synthesis, respectively. Media were supplemented with chloramphenicol (Cm 10 μg/ml), kanamycin (Kan 50 μg/ml), or ampicillin (Amp 100 μg/ml) when needed (Supplementary Table 1).

### 2.2. Molecular biology techniques

Primers used in this study are listed in Supplementary Table 2. All molecular biology techniques were performed following the manufacturer’s recommendations. Q5® High-Fidelity DNA Polymerase (New England BioLabs) and GoTaq DNA Polymerase (Promega, Madison, Wisconsin, USA) were used for the PCR reactions. 5′RACE experiments were performed with the 5′/3′ RACE kit (Sigma-Aldrich, Saint-Louis, Missouri, United States), using SP1 or R (retrotranscription), SP2 (PCR with anchor primers), and SP3 (sequencing) primers and poly-G oligonucleotides. In the case of uncertainty, poly-A tailing was also used. Purifications of PCR products were performed with NucleoSpin Gel and PCR Clean-up kit (Macherey-Nagel, Düren, Germany), and plasmid extractions were achieved using NucleoSpin Plasmid kit (Macherey-Nagel). Digestions were generated using restriction enzymes (Promega, Madison, Wisconsin, United States) and ligations with T4 DNA Ligase (New England BioLabs).

### 2.3. Construction of *Enterococcus faecalis* mutant strains

All mutant strains (Δ*nagY*, ∆*5’nagY*, Δ*hylA*) were constructed in *E*. *faecalis* V19 (Supplementary Table 1) using *E*. *coli* DH-5α as intermediate cloning host. pLT06 vector (Thurlow et al., 2009) was amplified with pLT06_1_bis and pLT06_2 primers, and flanking regions of the region to be deleted were amplified by PCR using oligonucleotides 1 and 2 for the upstream fragment, and oligonucleotides 3 and 4 for downstream fragment (Supplementary Table 2). Primers 1 and 4 have overlapping tails compatible with pLT06_1_bis and pLT06_2 primers, and primers 2 and 3 have overlapping tails compatible with each other. Cloning was performed using the *in vivo* recombination method (Huang et al., 2017). Deletion was obtained by double crossing over, as previously described (Thurlow et al., 2009), and was checked by PCR using primers 5 and 6 (Supplementary Table 2). Gene deletion and absence of point mutations susceptible to change phenotypes of deleted strains were checked by whole genome sequencing as described in Supplementary Material and Methods, and a summary of the variant detection analysis is listed in Supplementary Table 3.

### 2.4. Total RNA extraction

Cultures of 10 ml were performed at 37°C in carbon depleted medium cdM17 (La Carbona et al., 2007) supplemented with the appropriate sugar to OD_600_ 0.5. Cells were pelleted and lysed using a FastPrep device (MP Biomedicals, Illkirch Graffenstaden, France). RNA extraction and purification were achieved with TRIzol Reagent (ThermoFisher) and chloroform/isoamyl alcohol separation before using Direct-Zol RNA Miniprep kit following the manufacturer’s recommendations (Zymo-Research, Irvine, Californie, United States). RNAs were quantified using Nanodrop™ 2000 (ThermoFisher) and their quality was checked by electrophoresis.

### 2.5. RT-PCR and RT-qPCR

Reverse Transcription for RT-PCR and RT-qPCR assays were performed using QuantiTect Reverse Transcription kit (Qiagen) with L/R oligonucleotides and random primers, respectively (Supplementary Table 2). The GoTaq qPCR Master Mix (Promega) was used for qPCR, as well as the C1000™ Thermal Cycler (Bio-Rad) apparatus, using the following conditions: 95°C for 3 min, and 40 cycles at 95°C for 15 sec, 60°C for 1 min. Normalization was performed using the *gyrA* reference gene. Standard curves of each gene and qPCR efficiency were obtained using genomic DNA of the *E*. *faecalis* V19 strain.

### 2.6. *In vitro* production of RNA

RNA synthesis was achieved with the pTOPO plasmid and *in vitro* synthesis. DNA regions of interest were amplified by PCR using primers containing tails overlapping with pTOPO: topo85_FP1 and topo85_RP1 for 5’*nagY*, topo5’3023_FP1/topo5’3023_RP1 for 5’*hylA*, and topo65_FP1 and topo65_RP1 for SRC65 (Supplementary Table 2). Plasmid was amplified with topo_FP2 and topo_RP2 primers. PCR products were inserted into the plasmid with NEBuilder HiFi DNA Assembly Cloning Kit (New England BioLabs), and products used to transform *E*. *coli* TOP10. RNA production was performed on the resulting plasmid, linearized with the *Spe*I restriction endonuclease, using MAXIScript™ T7 *in vitro* Transcription Kit (Invitrogen, Carlsbad, California, United States). Unincorporated nucleotides were eliminated by ammonium acetate/ethanol precipitation, as recommended on the kit protocol, and RNA were quantified with Nanodrop™ 2000 (ThermoFisher).

### 2.7. Synthesis and purification of recombinant NagY protein

The *nagY* gene was amplified with primers ef1515_pQE70_*Sph*I and ef1515_pQE70_*Bgl*II carrying restriction sites (Supplementary Table 2). pQE70 plasmid and PCR product were digested with *Sph*I and *Bgl*II enzymes, ligated together, and used to transform *E*. *coli* M15 pRep4. The bacteria obtained were grown at 37°C with agitation in Terrific Broth medium supplemented with kanamycin and ampicillin until OD_600_ 0.5. Transcription induction was triggered with 0.5 mM isopropyl β-D-1-thiogalactopyranoside (IPTG) during 4 h at 37°C under agitation. NagY protein was purified with Protino Ni-NTA Agarose kit according to the manufacturer’s instructions (Macherey-Nagel), and desalted with PD10 columns (GE Healthcare, Chicago, Illinois, United States). Protein concentration was determined with the BCA test (ThermoFisher Pierce), and its purity checked on 12.5% SDS-PAGE and by mass spectrometry.

### 2.8. MicroScale thermophoresis (MST)

Recombinant NagY protein was labeled using the His-Tag Labeling Kit RED-tris-NTA 2^nd^ generation Monolith (Nanotemper Technologies, München, Germany) following the manufacturer’s recommendations, and diluted in ES-Buffer (10 mM Tris pH8.0, 40 mM NaCl, 10 mM KCl, 1 mM MgCl_2_, 0.05% Tween-80) at a final concentration of 25 nM. Before the assay, RNAs were heated for 5 min at 70°C and slowly cooled down at room temperature to allow a proper formation of secondary structures. A series of 1:1 dilution of RNAs were prepared in order to obtain a RNAs concentration ranged from 0.015 nM to 492 nM (16 points). Then each tube prepared with this 16 RNAs different concentration is mixed with the labeled protein (1:1), and filled into capillaries and introduced into the Monolith NT.115^Pico^ instrument (NanoTemper Technologies). Data of at least three independently pipetted measurements were analyzed (MO.Affinity Analysis software version 1.5.41, NanoTemper Technologies). The data were fitted using the law of mass action from GraphPad Prism version 5, and MicroScale thermophoresis (MST) figures were generated using MO.Affinity Analysis.

### 2.9. Biofilm study

Overnight cultures in GM17 of *E*. *faecalis* were adjusted to OD_600_ of 0.2 in fresh M17 supplemented with 2% glucose. One hundred microliters of the bacterial suspension were inoculated into CytoOne polystyrene microwells plate coated with 1 μg/ml of hyaluronic acid (Sigma-Aldrich), chondroitin sulfate (Carl Roth, Karlsruhe, Germany), or heparin sodium (ThermoFisher). After 24 or 48 h of incubation at 37°C, the plates were washed with 0.9% NaCl to remove unbound bacteria. Each well was then stained with 0.1% (wt/vol) crystal violet for 15 min at room temperature. Wells were then rinsed two times with 0.9% NaCl. Adherent cells were dissolved in 30% acetic acid, and the OD_550_ was measured using a microplate reader Nano Tecan (Life Science). At least three experiments were performed for each condition.

### 2.10. Detection of glycosaminoglycans-degrading activity

Detection of GAGs-degrading activity were performed as previously described (Kawai et al., 2018), with the following modifications. Overnight cultured cells were centrifuged at 4,500 rpm for 10 min, washed with 1 ml of 0.9% NaCl or GM17, and resuspended in the saline or GM17. The volume (X μl) of the saline or GM17 was calculated by the following formula: X = 200 × OD_600_. Cell suspension was then spotted at the center of the GAG minimal plate [0.2% GAG (hyaluronic acid, chondroitin sulfate, or heparin sodium), 0.1% yeast extract, 0.1% Na_2_HPO_4_, 0.1% KH_2_PO_4_, 0.01% MgSO_4_·7H_2_O, and 1.5% agar] with BSA at 1% and cultured at 37°C for 7 days. After cell growth, 1 ml of 2 M acetic acid was added to form a complex with the remaining GAG and BSA as white precipitates.

### 2.11. Virulence study on a *Galleria mellonella* model

Infection assays were performed on *Galleria mellonella* larva as previously described (Benachour et al., 2012). Bacteria were inoculated with a dose of 2 × 10^6^ CFU to 15 caterpillars per strain for each experiment. At least three experiments were performed for each condition. *G. mellonella* survival was followed from 16 h post-infection and during 24 h.

### 2.12. Phylogenomic analyses

#### 2.12.1. Genome samples, annotation, and quality assessment

Genomes were retrieved from the NCBI website (https://ftp.ncbi.nlm.nih.gov/genomes/, last accessed November 17,2021). DNA sequences of 2064 *E*. *faecalis* genomes were extracted and the GTDB database was used to select a set of 81 genomes of *Enterococcaceae* species, annotated as representatives (https://gtdb.ecogenomic.org/; Parks et al., 2022), and including the ATCC 19433 strain genome for *E*. *faecalis* (Supplementary Table 4). All genomes were annotated with *Prokka* (version 1.14.6; Seemann, 2014), and protein domains were annotated with the *hmmscan* program of the HMMER suite (version HMMER 3.1b2; Eddy, 2011). Three filters were used to ensure the quality of the genomes selected. First, we identified genomes for which the number of coding sequences predicted by *Prokka* is outlier. Then, the quality assessment of *E*. *faecalis* genomes was performed using *CheckM* (version 1.1.3; Parks et al., 2022). Finally, the *Mash* software (version 2.3; Ondov et al., 2016) was used to identify genomes incorrectly classified as *E*. *faecalis*. The intersection of the lists of genomes retained leads to a set of 1,949 *E*. *faecalis* strains.

#### 2.12.2. Pan-genomes

The core genome was used for inferring phylogenetic trees and the accessory genome to study the adaptation of different strains to biotope (Tettelin et al., 2005). To analyze the pan-genomes, phylogeny enhanced pipeline for pan-genome (*PEPPAN*) has been used for the *Enterococcus* species, and *Panaroo* (version 1.2.10) for *E*. *faecalis* strains (Supplementary Material and Methods; Tonkin-Hill et al., 2020; Zhou et al., 2020).

#### 2.12.3. *Enterococcaceae* species and *Enterococcus faecalis* strain trees

A maximum likelihood tree has been inferred based on the concatenation of 526 clusters of orthologous genes (OGs) identified by *PEPPAN* and aligned with *mafft* (*−-localpair --maxiterate 100*; Katoh and Standley, 2013). The strains V583 and OG1RF were added to the 81 reference genomes. The columns of the alignments that had a high deletion frequency were removed with *trimal* (*−gt 0*.*2*; Capella-Gutiérrez et al., 2009). The final alignment included 83 sequences with 171,295 columns and 99,042 phylogenetic informative sites. The tree was computed with *IQ-TREE* 2.2.0 (Minh et al., 2020) with the selected model Q.LG + F + R8, and branch support values were determined using ultra-fast bootstrap approximation (*ufboot*) and SH test (*alrt*) with 1,000 replicates. The tree was rooted on the *Enterococcus* sp. from Marseille-P2817 strains (Supplementary Figure 1). The *E*. *faecalis* strain tree was calculated from the concatenated alignments of the OG clusters identified by *Panaroo* and the tree constructed as described for *Enterococcacceae* but with *IQ-TREE* fast version (Minh et al., 2020) and the general time reversible model (GTR). Tree rooting was ascertained by adding the genomes of *E*. *caccae* and *E*. *rivorum*, which are the closest species to *E*. *faecalis* on the *Enterococcaceae* species tree (Supplementary Material and Methods; Supplementary Figure 1).

#### 2.12.4. NagY, NagE, and HylA orthologs

To identify the set of *Enterococcaceae* proteins that were orthologous to NagY, NagE, and HylA from *E*. *faecalis* V583, we used the OG clusters calculated with the *PEPPAN* pipeline and the genetic context conservation (Supplementary Table 5). The genetic context of the genes is obtained by extracting the gene positions from the *Prokka* GFF files. The annotation files were prepared with in-house scripts and the trees were annotated and visualized with the online tool Interactive Tree Of Life (iTOL v6; https://itol.embl.de; Letunic and Bork, 2019). The Pfam domain annotation of the proteins was predicted with the *hmmscan* program (Eddy, 2011). *GeneRax* (version 2.0.4; Morel et al., 2020) was then used to infer rooted family tree directly from the multiple sequence alignment and a rooted species tree. Each dataset was aligned with *mafft* and the amino acid substitution model that best fit the data were selected with *modeltest-ng* (v0.1.7; Darriba et al., 2020). In addition to the protein family root tree, the program returns statistics on the events predicted by the reconciliation (speciation, speciation+loss, duplication, and transfer). For the identification of OGs in *E*. *faecalis* strains, the V583 protein sequences were used as query with *mmseqs2* (Steinegger and Söding, 2017) on the entire *E*. *faecalis* proteomes annotated by *Prokka*. Next, we identified the *Panaroo* OGs to which significant hits belong and extracted all proteins from each OG.

#### 2.12.5. Identification of conserved motifs

The conserved motifs on *Enterococcaceae* 5’*nagY* were identified with *MEME* 5.3.0 (Bailey and Elkan, 1994). A secondary structure search was performed with *RNAfold* 2.4.14 from the Vienna RNA package (Lorenz et al., 2011) and validated with *rLocARNA* 2.8.ORC8 software (Will et al., 2012) that simultaneously folds and aligns the input sequences. The alignment obtained with *rLocARNA* was used to construct a covariance model that combines primary sequence conservation and secondary RNA structure (*cmbuild* and *cmcalibrate* from the Infernal package 1.1.4; Nawrocki and Eddy, 2013). The *cmsearch* (Infernal package 1.1.4) was used to search for these patterns in the DNA sequences.

## 3. Results

### 3.1. Identification and phylogenetic distribution of the *nagY* and *nagE* orthologs in *Enterococcaceae*

**Figure 2:**
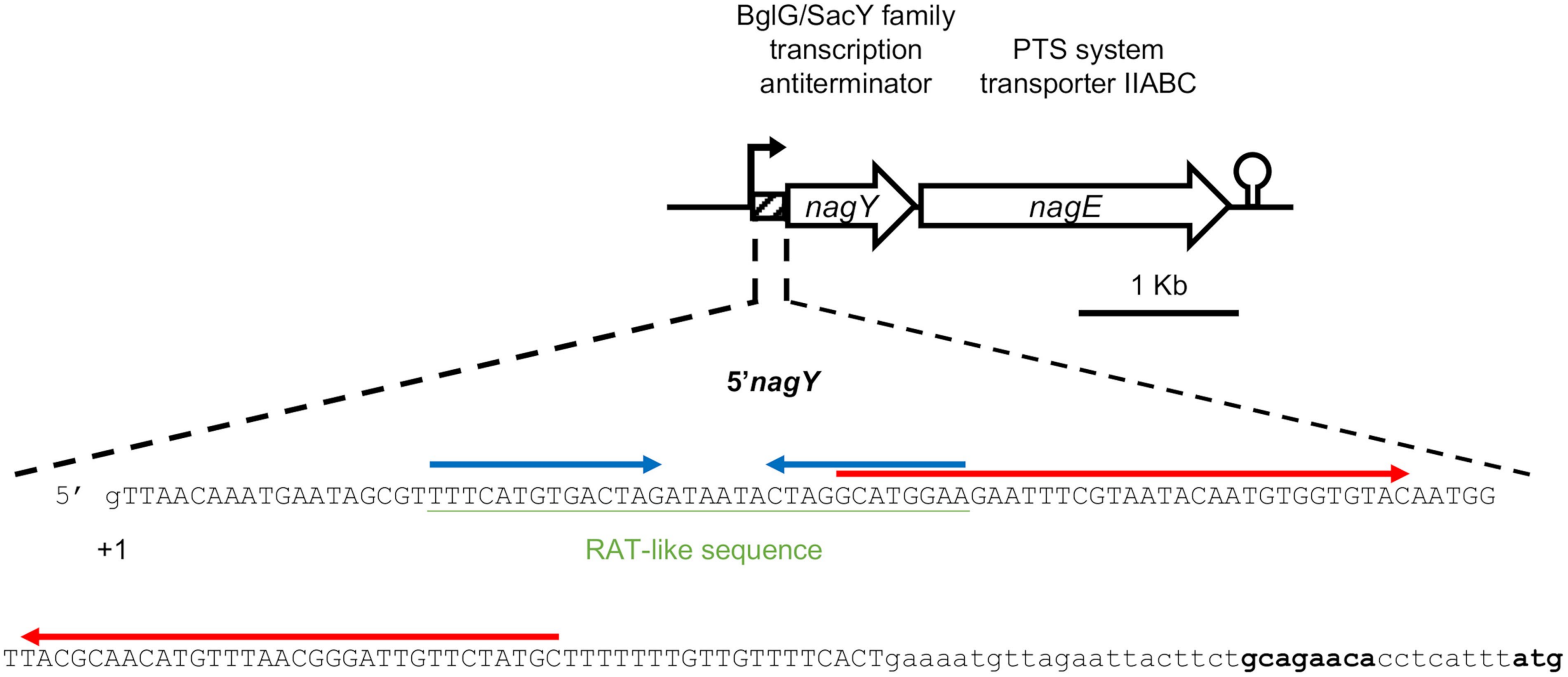
Representation of the *nagY-nagE* operon and 5’*nagY* sequence. Transcription start (+1), ribosome binding, and translation start (in bold) sites are indicated. The sequence deleted in the Δ*5’nagY* mutant is in capital letters. The RAT-like sequences are underlined in green, and the terminator and the antiterminator identified are overligned by inverted arrows in red and blue, respectively.

The *ef1515* gene in *E*. *faecalis* V583 is annotated as encoding a BglG/SacY family antiterminator, and we renamed it *nagY* in reference to *sacY*, and its following gene *nagE* (Mao et al., 2009). The EF1515 (EF_RS07320 in the new nomenclature) protein presents 36% identity with SacY from *B*. *subtilis,* and 31% amino acid identity with BglG from *E*. *coli* (*blastP* alignment). This gene is followed by the *ef1516* (EF_RS07325) gene (Figure 2), recently renamed *nagE* on V583 genome, encoding a NAG specific EIICBA PTS transporter (Keffeler et al., 2021b). Thus, the NagY and NagE proteins belong to multigene families. In order to unambiguously identify the orthologous genes encoding these proteins in the *Enterococcaceae*, we performed a phylogenomic analysis of these families.

**Figure 3:**
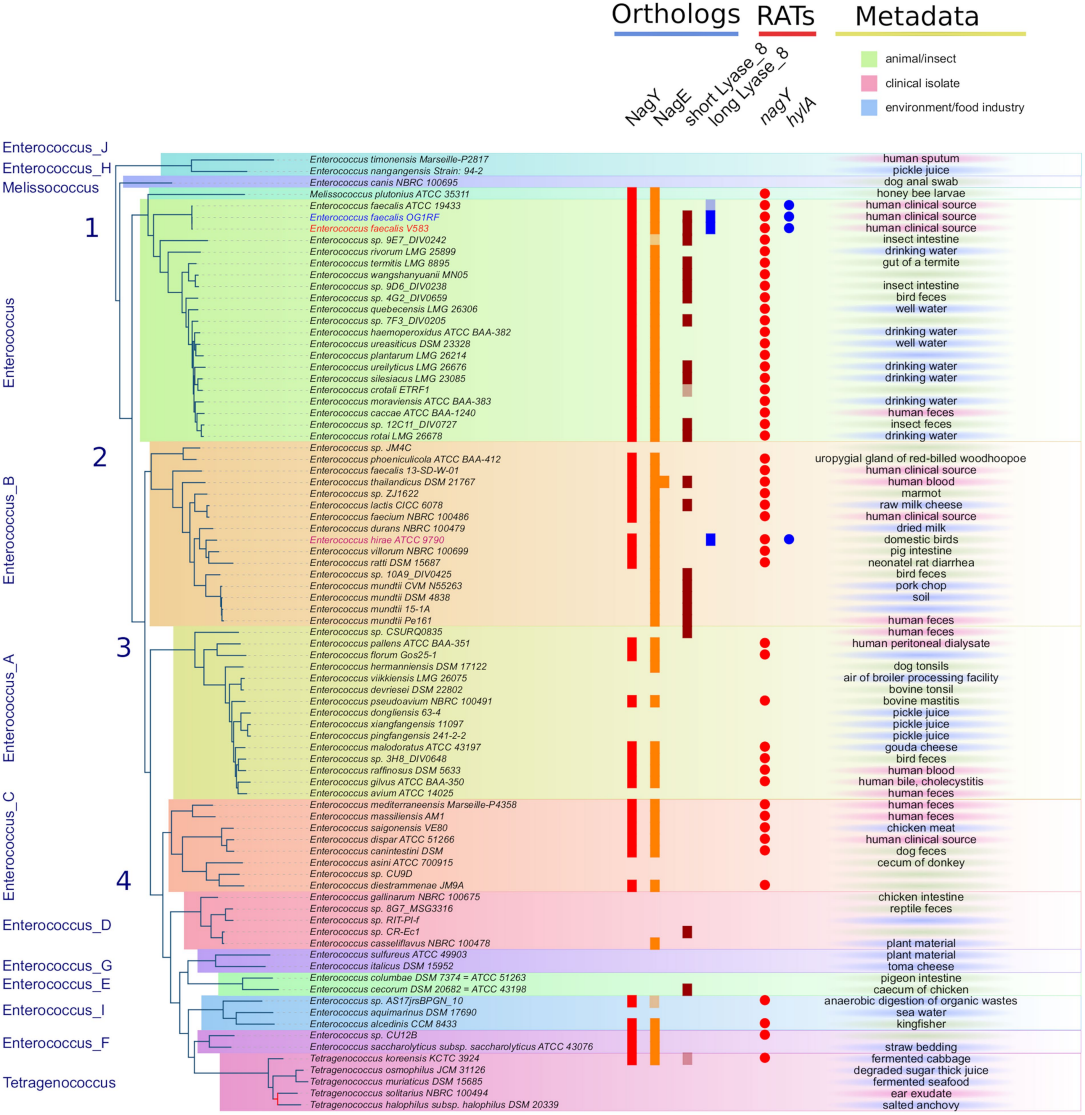
Distribution of NagY, NagE, and HylA protein families in *Enterococcaceae* genomes. First panel on the left: The *Enterococcaceae* species tree inferred with the 526 orthologous gene (OGs) clusters present in at least 95% of the genomes. The tree was rooted with Marseille-P2817 strains (Supplementary Material and Methods). The 13 family genera of *Enterococcaceae* described in the GTDB and the four phylogenetic groups described in Lebreton et al. (2017) were reported. The tree is perfectly resolved except for one branch of the *Tetragenococcus* subtree colored in red (13.1/54, *ufboot/alrt* supports). Second panel in the middle-left (“Orthologs”): distribution of orthologous proteins to NagY, NagE, and short and long Lyase_8 forms. Lightened colors indicate the presence of pseudogenes. Third panel in the middle-right (“RATs”): occurrence of RAT sequence in front of *nagY* and *hylA* genes. Last column on the right (“Metadata”): metadata available in GenBank files of the analyzed genomes.

The *PEPPAN* pipeline (Zhou et al., 2020) has been used to analyze the pan-genomes of the 81 *Enterococcaceae* reference genomes. *E*. *faecalis* V583 *nagY* and *nagE* belong to two OG clusters composed of 47 and 54 members. Both genes appear to be present in the majority of *Enterococcaceae* studied (Figure 3). They are absent in the genera Enterococcus_J, _H, _E, and _G and weakly represented in the genus Enterococcus_D and in Tetragenococcus. Note that the outgroup position of Enterococcus_J and Enterococcus_H suggests that these genes were absent in the last common ancestor (LCA) of *Enterococcaceae*. Two *nagE* paralogs are present in *E*. *thailandicus* DSM 21767 and a *tblastn* search, with the candidate genes as query, identifies two *nagE*-like sequences in two genomes (*E*. sp. 9E7_DIV0242 and *E*. *sp*. AS17jrsBPGB_10) that were not annotated by *Prokka* (Figure 3). Chromosomal neighborhood analysis of the *nagY* genes reveals, in all cases, the presence of a downstream *nagE* gene. However, in eight genomes, a *nagE* gene is present without its *nagY* partner. Five genomes belong to a subtree composed of *E*. *sp*. 10A9_DIV0425 and four *E*. *mundtti* genomes (Figure 3) suggesting that the *nagY* gene may have been lost in their LCA. One of the two *E*. *thailandicus* DSM 21767 paralogs does not have a *nagY* gene in its chromosomal neighborhood. Note that *nagE* genes encode proteins with the EIICBA architecture as PtsG of *B*. *subtilis* (and *B*. *cereus*) while BglF from *E*. *coli* has EIIBCA domain order.

In addition to gene losses, *GeneRax* predicted that horizontal gene transfers (HGT) may have occurred, with a higher frequency for *nagE* (17 HGTs for 54 genes) than for *nagY* (7 HGTs for 47 genes; Supplementary Figures 2A,B). Some HGTs may have occurred between genomes belonging to different genera, as revealed by the splitting of these genera on the protein trees. It should be noted that no conservation of gene neighborhoods was found in the genomes studied. Only a majority of strains of the genus *Enterococcus* present a conserved genetic context, with the notable exception of the *E*. *faecalis* V583 strain (Supplementary Figure 2).

To determine the extent to which the orthologous gene pair *nagY-nagE* is present in *E*. *faecalis* strains, we searched the 1949 genomes for orthologs of both gene products. The NagY and NagE proteins were identified in 99.84 and 99.59% of the genomes, respectively, and both are present in 99.38%. Missing genes are due to incomplete genome assemblies.

### 3.2. Characterization of the *nagY-nagE* operon and its regulation

**Figure 4:**
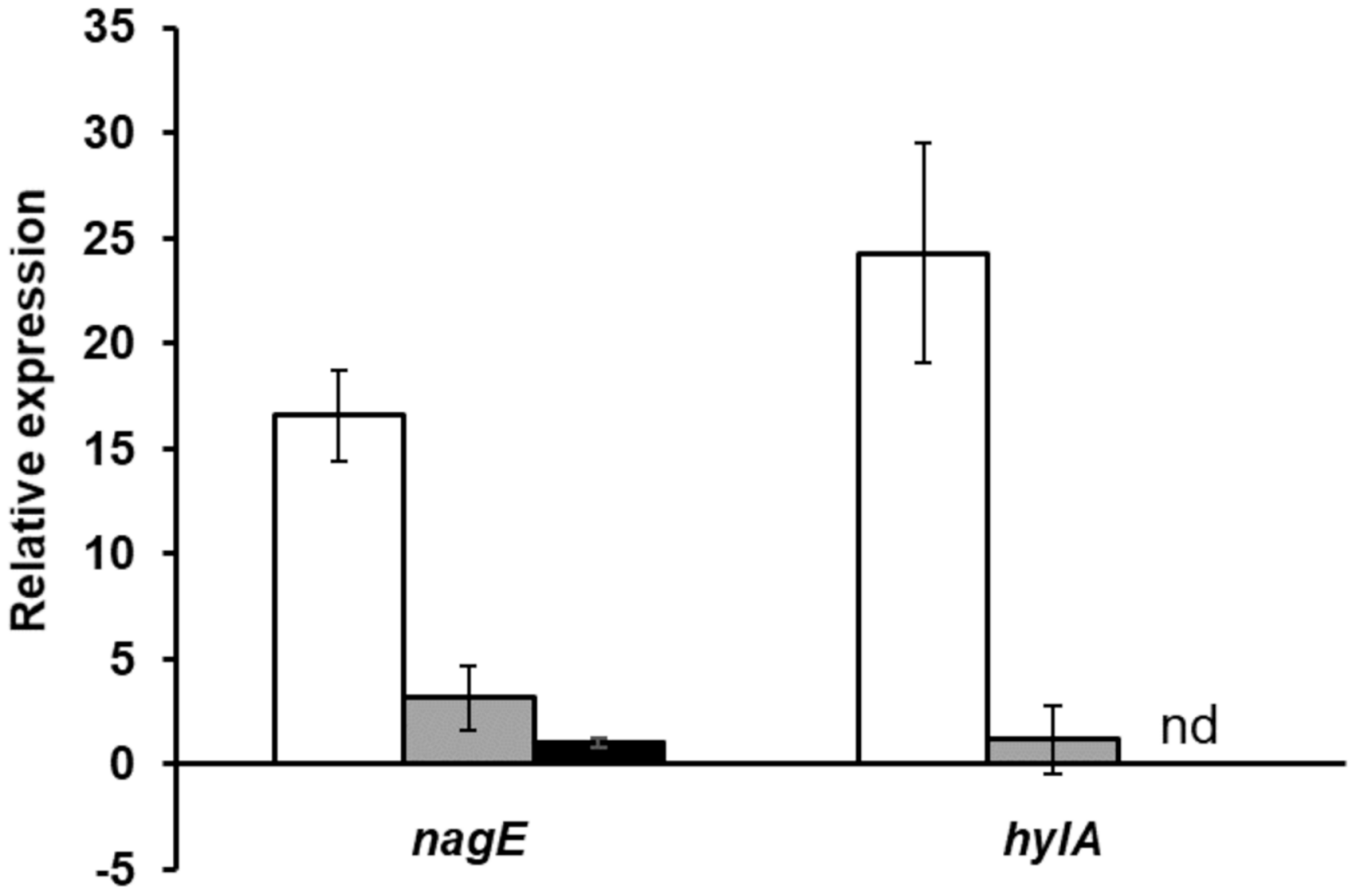
Study of *nagE* and *hylA* induction of expression in presence of NAG compared to glucose condition. The *nagE* and *hylA* gene expression in WT (white), Δ*nagY* (gray), and Δ*5’nagY* (black) strains were revealed by RT-qPCR, with RNA purified from culture in presence of NAG as sole carbon source compared to glucose condition. Error bars represent the standard error of triplicate measurements. nd: not determined.

We experimentally characterized the *nagY-nagE* operon (represented in Figure 2) by confirming the co-transcription of these two genes by RT-PCR (Supplementary Figure 3). The induction of the *nagY-nagE* expression in presence of NAG was then revealed by RT-qPCR targeting *nagE* (Figure 4). This gene is overexpressed in WT strain in presence of NAG as sole carbon source, with a fold-change (FC) of 16.6-fold (±2.15) compared to glucose conditions (*p* < 0.001), and this induction is almost completely abolished in the Δ*nagY* strain (FC = 3.1 ± 1.55, *p* < 0.001).

To investigate the role of NagY in its own operon regulation, we firstly identified the transcription start site by 5′RACE-PCR assay. Thus, consistent with previous studies (Innocenti et al., 2015; Muller et al., 2015; Michaux et al., 2020), the starting base of the RNA was confirmed to be located 172 pb before the predicted initiation codon (Figure 2; Supplementary Figure 4A), showing the existence of a long 5′UTR (named 5′*nagY*). The search of RAT sequence based on the consensus defined in preceding work in *E*. *coli* and *B*. *subtilis* (Aymerich and Steinmetz, 1992; Tortosa et al., 2001; Gordon et al., 2015) allowed the identification in the 5′*nagY* of an imperfect inverted repeat with a low sequence conservation with the consensus sequence (Figure 2). A search performed with *MEME* (Bailey and Elkan, 1994) in the upstream regions of *nagY* orthologs reveals the presence of two conserved motifs. The first motif covers the region overlapping the putative RAT sequence identified above in *E*. *faecalis* V583. The second motif, located downstream of the first motif, is less well conserved but is characterized by a terminal T-rich region (Supplementary Figure 5). These motifs are conserved with their relative positions upstream of the 46 *nagY* sequences of *Enterococcaceae*, with the exception of *E*. *saccharolyticus* (Figure 3). Alignment of the first motif with *rLocARNA* (Will et al., 2012) reveals a conserved stem loop with a two-nucleotide bulge (Supplementary Figure 5A). The stem bases have undergone a large number of compensatory mutations to maintain this secondary structure. The region bounded by the two conserved *MEME* motifs was extracted from the 5’*nagY* for the different genomes. The *rLocARNA* predicts, in all sequences, the presence of a large stem loop of variable length that ends in a T/U-rich region, a structure typical of an independent rho terminator (Supplementary Figure 5B). The foot of this structure overlaps the end of the RAT region (common consensus sequence: GCRUGGA). These two structures are therefore mutually exclusive (Supplementary Figure 5C). This competition between the two structures is typical of what has been observed for BglG/SacY antiterminators. A covariance model was constructed with the alignment obtained with *rLocARNA* and was used to identify the RAT-terminator motif with a high specificity in genomic sequences (Figure 3).

**Figure 5:**
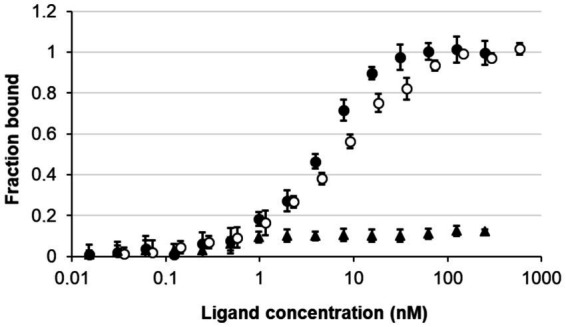
Study of the interaction of NagY on the 5’UTR RNA target genes. MST dose response curves for interaction between NagY labeled protein and 5’*nagY* (black circles), 5’*hylA* (empty circles) RNA (ligand), and SRC65 sRNA (used as negative control; black triangles). Error bars represent the standard error of triplicate measurements.

To confirm that the *nagY-nagE* transcription-antitermination mechanism is similar to the SacY model in *B*. *subtilis*, the direct interaction between NagY and 5’*nagY* was studied by MST. The purified protein was incubated in presence of the *in vitro* produced 5’*nagY* RNA. As shown in Figure 5, we observed a dose–response binding of NagY on 5’*nagY*, with a Kd of 4.18 nM (±0.42 nM). The SRC65 sRNA (Shioya et al., 2011; Salze et al., 2020b) was used as negative control and did not show any interaction with NagY. These results indicate that NagY has a high affinity for its 5′UTR and regulates both NagE and its own expression by its binding on RNA 5’*nagY*. Consequently, binding of NagY to RATs can lead to the opening of the hairpin base and therefore make the transcription terminator ineffective.

To confirm that these sequences are involved in the *nagY-nagE* NAG-dependent induction, a deletion of 5’*nagY* region overlapping both structures was constructed in the WT strain (Figure 2), and the expression of *nagE* in this mutant was determined by RT-qPCR (Figure 4). No NAG-dependent induction was observed when we compared NAG to glucose culture conditions, but *nagE* is deregulated whatever culture conditions are, with FC of 33.2 and 34.8 in presence of glucose and NAG, respectively (Supplementary Figure 6). Considering the role of NagE in the NAG transport, the operon could potentially be regulated by catabolic repression. The expression of the operon was also followed in the presence of glucose and NAG, but no difference of expression was observed compared to the condition with NAG only (Supplementary Figure 6). These results establish that the *nagY-nagE* operon expression is not under the control of the catabolic repression.

### 3.3. Identification of a new NagY target gene encoding a polysaccharide lyase

As regulator binding on nucleic acid, NagY could potentially regulate other genes expression by recognizing a conserved sequence. To identify potential NagY targets, we searched for RAT/terminator motif with *cmsearch* software (Infernal package; Nawrocki and Eddy, 2013) in the *E*. *faecalis* V583 genome. We obtained two hits, the highest upstream of the *nagY* gene, and the second upstream of the e*f3023* (e*f_rs14340*) monocositronic gene, named *hylA, w*hich shares 88% identity with the *nagY* RAT-like sequence. This suggests that *hylA* possesses a 5’UTR (named 5’*hylA*) on which NagY could potentially bind to regulate the expression of this gene. We confirmed the existence of a 5’*hylA* of 193 pb long by 5’RACE-PCR (Supplementary Figure 4B), and the interaction between NagY and this RNA was studied by MST (Figure 5). This assay highlighted a binding of the protein on 5’*hylA* RNA, with a lower affinity than with 5’*nagY* (7.46 ± 0.63 nM). Moreover, RT-qPCR assays showed that the expression of *hylA* depends on the presence of the *nagY* gene (Figure 4). These results are compliant with the hypothesis that NagY also regulates the expression of *hylA*, suggesting that *nagY*, *nagE*, and *hylA* belong to the same regulon and potentially the same carbohydrate consumption pathway.

**Figure 6:**
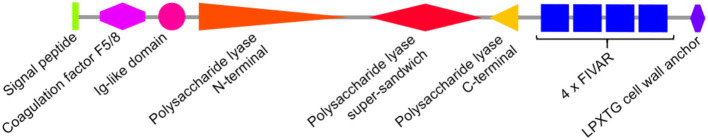
HylA protein of *Enterococcus faecalis*. The HylA protein is composed of a N-terminal signal peptide, a coagulation factor F5/8 domain (F5_F8_type_C PF00754), an Ig-like domain (Big_2 PF02368), enzymatic family 8 polysaccharide lyase domains (Lyase_8_N PF08124, Lyase_8 PF02278, and Lyase_8_C PF02884.17), a repetition of four FIVAR domains (Found In Various Architectures PF07554), and a LPXTG cell-wall anchor domain (Gram_pos_anchor PF00746). The C-terminal transmembrane and intracytoplasmic domains are not shown. The short version of Lyase_8 is solely constituted of lyase domains.

HylA was identified as a cell-wall anchored protein, annotated as a polysaccharide lyase 8 [Lyase_8_N (PF08124), Lyase_8 (PF02278), and Lyase_8_C (PF02884.17) domains, Figure 6]: this group of enzymes targets uronic acid-containing polysaccharides such as some GAGs (hyaluronate, chondroitin, or heparin) that are components of the ECM (Sillanpaa et al., 2004, 2009; Lombard et al., 2014). To our knowledge, the function and substrate of *E*. *faecalis* HylA are unknown, although it is annotated as hyaluronidase in KEGG database (Kanehisa et al., 2017) and was identified as a MSCRAMM family member mostly extracytoplasmic (Sillanpaa et al., 2004, 2009). In addition to a signal peptide and the lyase regions, HylA possesses other domains: (i) a F5/8 type C domain (discoidin domain) that is a major domain of many blood coagulation factors (F5_F8_type_C PF00754), (ii) a bacterial Ig-like domain (Big_2 PF02368) found in bacterial cell-adhesion molecule, mediating the intimate bacterial host-cell interaction (Kelly et al., 1999), (iii) FIVAR domains (Found In Various Architectures PF07554) mostly associated to binding domains in cell wall associated proteins, and (iv) a LPXTG cell wall anchor motif (Gram_pos_anchor PF00746) presents in virulence factors which are produced by Gram positive pathogens.

### 3.4. Identification and phylogenetic distribution of the *hylA* homologs in *Enterococcaceae*

We used the two largest conserved domains of the protein to identify homologous sequences in *Enterococcaceae* (PF08124, Lyase_8_N and PF02278, Lyase_8) and the simultaneous occurrence of these two domains was found in 27 proteins. These proteins also have the PF02884 Lyase_8_C domain. Three of them are significantly longer and have the same domain architecture as *E*. *faecalis* HylA, except for the number of FIVAR domains (Supplementary Figure 2C). The formers are referred to as short Lyase_8 and the latter as long Lyase_8. The *SignalP* program (Almagro Armenteros et al., 2019) predicts a standard secretory signal peptide in all proteins. Two proteins are very partial (155 AA for *T*. *koreensis* KCTC 3924 sequence and 131 AA for *E*. *crotali* ETRF sequence). Note that both strains of *E*. *faecalis* (V583 and OG1RF) have both types of proteins. *Prokka* does not annotate a *hylA* gene in the genome of *E*. *faecalis* ATCC 19433A, but a search with *tblastn* reveals the presence of a partial sequence similar to a gene encoding a long Lyase_8 (Figure 3). The long Lyase_8 protein found in the genomes of *E*. *faecalis* V583 and OG1RF strains is also found in the genomes of *E*. *hirae* ATCC 9790 (Figure 3).

*GeneRax* predicted that the *hylA* sequences of *E*. *hirae* and *E*. *faecalis* are originated from HGTs, but presumably from genomes that are not sampled in our study (Supplementary Figure 2C). *cmsearch* of the upstream region of the *E*. *hirae hylA* gene reveals the presence of the RAT-containing motif with the T-rich region just downstream, as observed in the 5’*nagY* sequence. This conservation suggests similar regulation of *hylA* by NagY in *E*. *hirae* and *E*. *faecalis* strains. It also has to be noted that RAT sequence in the 5’*hylA* is conserved only in the presence of the long Lyase_8 (Figure 3).

To better understand the origin of HylA in *E*. *faecalis*, we ran a *blastP* on the NCBI website with the EF3023 sequence. Even though many sequences are from *E*. *hirae*, sequences from *Lacticaseibacillus* genomes and from different species of *Listeria*, *Staphylococcus*, and *Streptococcus* are found. If some of them are partial, others like those of *S*. *agalactiae* or *L*. *monocytogenes* have a domain organization similar to those of *E*. *faecalis* sequences. The high sequence conservation between these distant species suggests recent HGTs.

**Figure 7:**
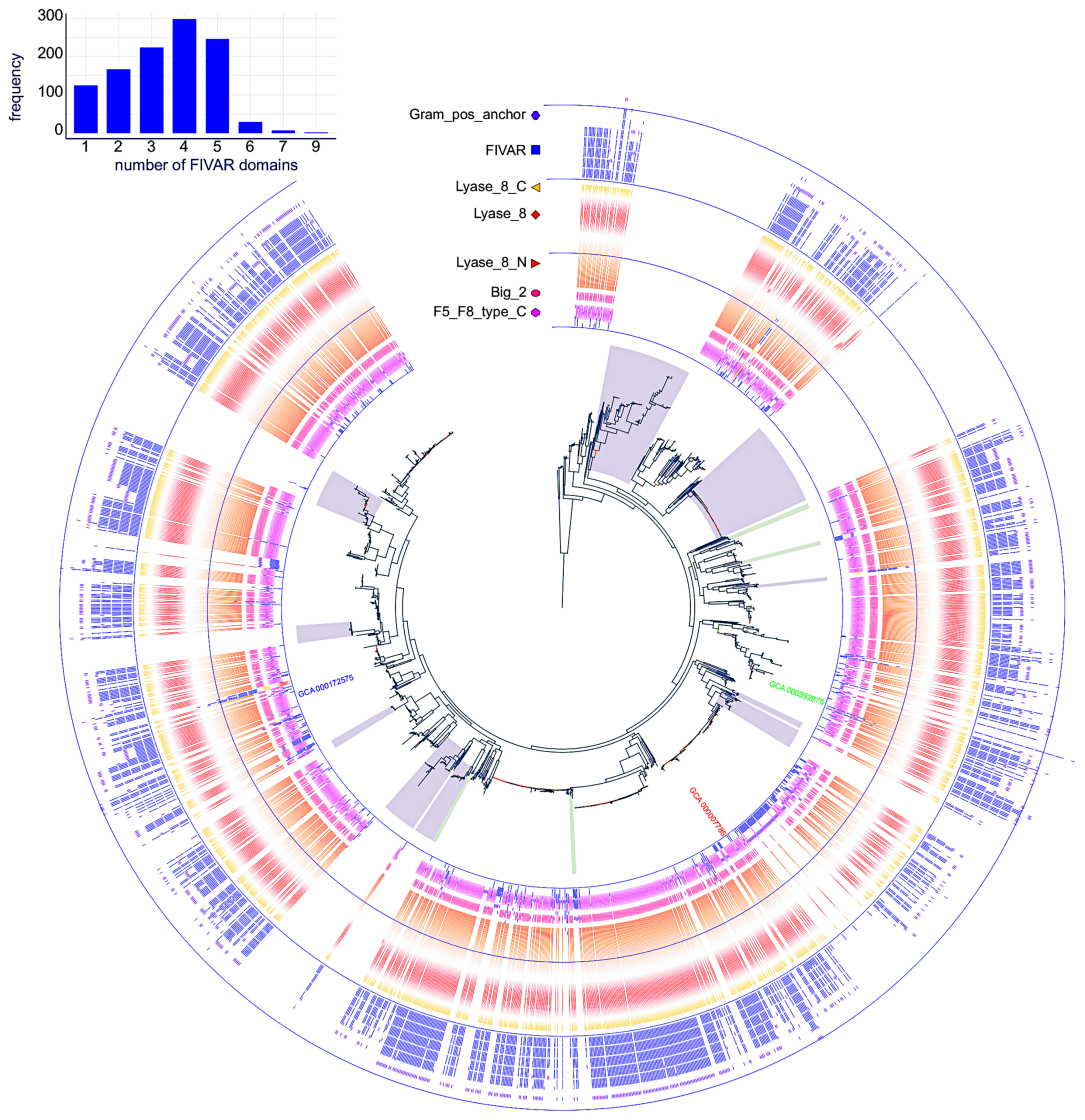
Distribution of HylA protein in *Enterococcus faecalis* genomes. The tree of the 1949 *E*. *faecalis* strains was inferred with *IQ-TREE* from the concatenated alignments of the orthologous gene clusters identified by *Panaroo*. Branch support, indicated by a color gradient (red to blue), was estimated with the SH approximate likelihood ratio test. The tree was rooted by adding the genomes of *E*. *caccae* and *E*. *rivorum*, which are the closest species to *E*. *faecalis* on the *Enterococcaceae* species tree. The location of strains V583 (GCA_000007785), OG1RF (GCA_000172575), and ATCC 19433 (GCA_000392875) on the tree are highlighted. The ring shows the organization of the Pfam domains of the proteins, with the same color code as in Figure 6. The absence of domains indicates the absence of the *hylA* gene in the strain. Subtrees where the *hylA* gene is absent are highlighted. The distribution of the number of FIVAR domains is plotted in the upper left corner.

To determine the extent to which the orthologous gene *hylA* is present in *E*. *faecalis* strains, we searched for occurrences of *hylA* in the 1949 *E*. *faecalis* proteomes. We identified 1,520 occurrences among which 458 sequences are partial with a length of their gene shorter than 3,000 nt (Figure 7). The *hylA* genes appears to be well distributed in *E*. *faecalis* strains, however we can observe its absence in some closely related species forming subtrees on the species tree, suggesting that it has been lost in their LCA. Similarly, we can note that partial sequences are found in genomes closely related on the species tree, which could indicate pseudogenization of these genes for subsets of related genomes. These *hylA* genes encode HylA protein but with a variable number of FIVAR domain(s) (from 1 to 9, but centered on four copies). The short version of the protein is present in 1,243 *E*. *faecalis* proteomes with 26 genes fissions.

### 3.5. Characterization of HylA

**Figure 8:**
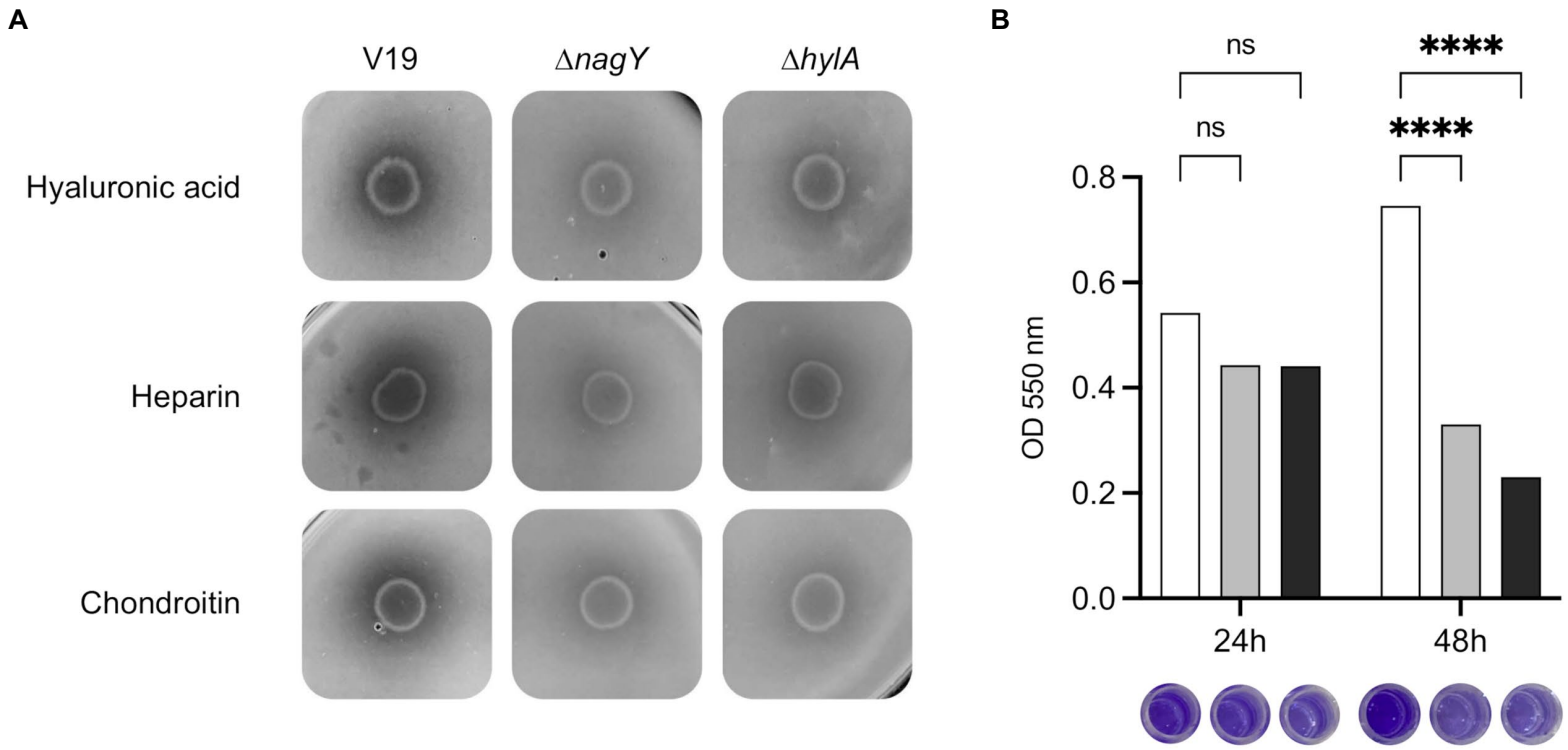
Involvement of NagY and HylA in GAG degradation and biofilm formation. **(A)** Degradation of GAGs (hyaluronic acid, heparin sodium, or chondroitin sulfate) by *Enterococcus faecalis* V19 WT, Δ*nagY*, and Δ*hylA* was studied after cells grown on GAG minimal plate with BSA and revealed by the addition of 2 M acetic acid, as described in the Materials and methods section. Halo formation is proportional to GAGs degradation. **(B)** Study of biofilm formation in WT (white), Δ*nagY* (gray) and Δ*hylA* (black) strains with the corresponding observation of crystal violet staining. Value of *p* < 0.0001 (Tukey’s multiple comparisons test). A coating with 1 μg/ml of hyaluronic acid was performed before biofilm formation. The experiment was realized three times.

To determine HylA functions, the Δ*hylA* mutant was constructed and characterized. We observed that this mutant was not affected in its growth in the presence of NAG as sole carbon source (Supplementary Material and Methods; Supplementary Figure 7). HylA has putative hyaluronidase domain, and hyaluronic acid is a polymer made up of alternating NAG and glucuronic acid residues linked by glycosidic bonds (Hynes and Walton, 2000), that could make the functional link between *hylA* and *nagY-nagE* operon. As shown on Figure 8A, Δ*nagY,* and Δ*hylA* cannot degrade this substrate compared to the WT strain, as well as heparin sodium and chondroitin sulfate, confirming that the encoding proteins are involved in the use of these GAGs.

Given the adhesin domains identified in the HylA protein, biofilm formation was assessed using the microtiter plate assay on a coating of hyaluronic acid, chondroitin sulfate, or heparin sodium (Figure 8B; Supplementary Figure 8). While no difference was observed after 24 h, a significant 2.3 and 3.2-fold decrease in crystal violet staining was observed after 48 h for the Δ*nagY* and Δ*hylA* mutants on microtiter plates coated with hyaluronic acid, respectively (Figure 8B), and 1.9/1.7 and 1.5/1.6-fold decrease on plates coated with heparin or chondroitin compared to the WT strain (*p* < 0.0001; Supplementary Figure 8).

**Figure 9:**
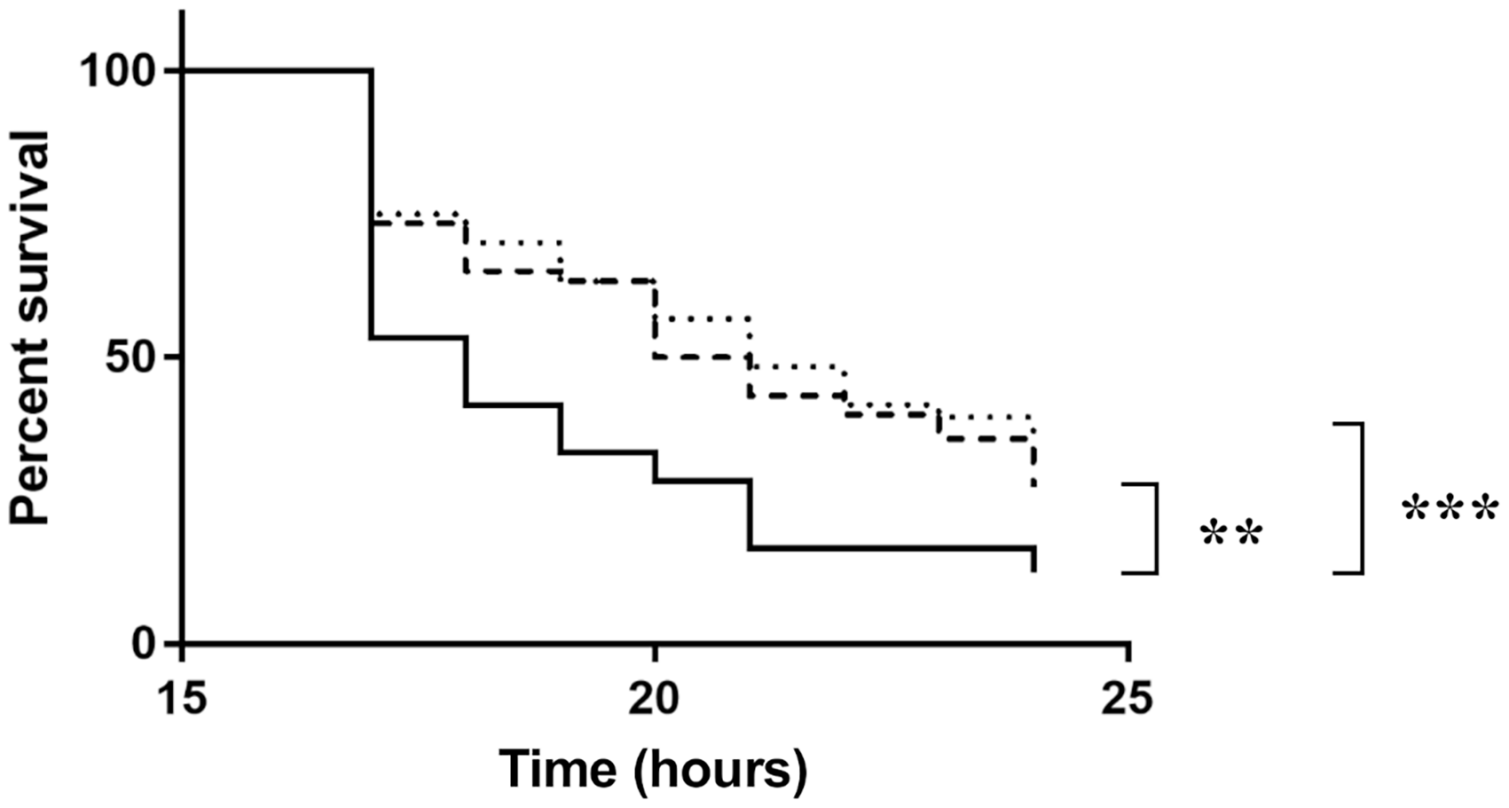
Survival of *Galleria mellonella* larvae infected by *Enterococcus faecalis* WT (solid line), Δ5’*nagY* (dashed line), or Δ*hylA* (dotted line) strains. The experiment was performed three times using 15 caterpillars per strain and per test. ^**^value of *p* < 0.01 comparatively to the V19 strain. ^***^value of *p* < 0.001 comparing to the V19 strain (log-rank test).

To investigate the role of NagY and HylA in virulence *in vivo*, we monitored *G. mellonella* larvae survival infected by WT, the two mutant strains and the 5’*nagY* mutant strain in which NagY is deregulated and overexpressed whatever conditions are (Figure 9; Supplementary Figure 9). The experiment showed that the deletion of Δ*nagY* does not affect larval mortality (Supplementary Figure 9). However, larvae infected by Δ*hylA* and ∆*5’nagY* strains showed a significant increase in survival relative to the parental WT strain (*p* < 0.001 and *p* < 0.01 respectively, Figure 9).

## 4. Discussion

In condition of equilibrium of the gastro-intestinal microbiota, *E*. *faecalis* is a subdominant species, but during dysbiosis (induced by antibiotic treatments for example), it overgrows and cross the intestinal barrier, giving rise to intestinal translocation and infection (Ubeda et al., 2010; Archambaud et al., 2019). During colonization, *E*. *faecalis* has to use specific mechanisms to adapt to a new environment and find out carbohydrates like mono- and polysaccharides or mucin components. NAG is one of the main nutrients used by bacteria during colonization (Chang et al., 2004). This sugar is found in large amount in the gastro-intestinal tract and a component of GAGs that made up the ECM.

Our study of 1949 *E*. *faecalis* strains revealed that the *nagY* and *nagE* were present in 99.84 and 99.59% of the genomes, respectively, and both are present in 99.38%. The absence of genes is likely to be due to incomplete genome assemblies. The analysis of a set of 81 reference genomes of *Enterococcaceae* shown that this gene pair is conserved in 47 genomes (Figure 3). Consequently, *nagY*-*nagE* is highly conserved in *E*. *faecalis*, and were lost or transferred frequently during the evolution of *Enterococcaceae,* illustrating the adaptation of genomes to the presence of NAG in their environment.

We observed that this operon is autoregulated thanks to NagY and its binding on 5’*nagY* RNA, implicating a *cis*-acting regulatory element containing a small secondary structure overlapping a rho-independent terminator. This terminator is conserved upstream of *nagY* in the *Enterococcaceae* as evidenced by the presence of compensatory mutations that preserve the structures (Figure 3; Supplementary Figure 5). Our results suggest that this first structure may interfere with the formation of the transcriptional terminator and therefore prevents early transcription termination. This structure was identified as the RAT sequences: the mechanism of NagY regulation in *E*. *faecalis*, and most likely in other *Enterococcaceae*, is consequently similar to the admitted model in *B*. *subtilis* and *E*. *coli* (Aymerich and Steinmetz, 1992; Amster-Choder, 2005). Transcription of *nagY*-*nagE* is constitutively initiated but stops at the terminator structure upstream of the coding region unless β-glucosides are present (Figure 1). Thus, the NagE PTS transporter allows the NagY antiterminator to sense carbohydrate source in the environment (Tortosa et al., 2001). NAG is then phosphorylated by NagE during its import into the cell (Keffeler et al., 2021b) and is directly used in glycolysis and metabolized. We showed that *nagY*-*nagE* is not submitted to catabolic repression. The consensus *cre* sequence WTGWAARCGYWWWC (Suárez et al., 2011) is indeed modified by the insertion of a nucleotide ATGAATAGCGTTTTC that probably interferes with catabolic repression. It has to be noted that the transcription unit controlled by SacY is also not subject to CCR (Stülke et al., 1998). Moreover, it was observed that *nagE* induction of expression is weak but still present in the absence of *nagY* when the strain is cultivated with NAG as sole carbon source (Figure 4; Supplementary Figure 6), suggesting another level of regulation. Whereas *nagY* gene in *E*. *faecalis* does not appear to be directly involved in pathogenesis in our caterpillar model with the ∆*nagY strain*, the observation of a significant increase in survival following infection with the ∆5’*nagY* (when NagY is constitutively expressed) highlights the involvement of the antiterminator in virulence (Figure 9). Its homolog in *L*. *monocytogenes* was also demonstrated to be a virulence factor (Abdelhamed et al., 2019). NagY is consequently supposed to be associated to pathogenesis, even if no clear correlation between the presence of *nagY* gene and clinical isolates origin was found (Figure 3).

NagY not only regulates the expression of its own operon, but also the *hylA* gene, encoding a hyaluronate lyase HylA enable to provide nutrient source from GAGs. Consequently, this hyaluronidase represents an advantage for nutrient recovery *in host* and infection process of *E*. *faecalis*. However, this activity is very low in our culture condition since no growth can be observed with GAGs like hyaluronic acid, chondroitin, or heparin as sole carbon source (Supplementary Figure 7), but GAGs degradation can be observed (Figure 8A). Previous report also showed that *E*. *faecalis* slightly degrades heparin (Kawai et al., 2018). Indeed, enterococci show little ability to degrade GAG, and use preferentially unsaturated GAG disaccharides produced by other bacteria in human gut microbiota (Kawai et al., 2018). Cross-feeding by anaerobes is by the way considered to be the major actor of polysaccharide degradation: the ability of enterococci to utilize such nutrients *in vivo* would obviously be dependent on their potential to compete effectively for them with members of the microbiota. In this context, HylA could be used as a backup to favorize *E*. *faecalis* survival and the competition with other microorganisms in gastro-intestinal microbiota. Contrary to *nagY-nagE* operon, it was shown that *hylA* gene expression is under the control of the RpoN sigma factor and CCR, suggesting a multifactorial regulation of this gene (Keffeler et al., 2021a). Thus, the *nagY, nagE*, and *hylA* genes could be involved in the adaptation of *Enterococcaceae* through the use of different carbohydrate sources. As hyaluronidases, *i.e.*, endolytic glycoside hydrolases, HylA protein would be complementary to EfChi18A-EfCBM33A and EndoE to retrieve NAG from environment, described in a recent study by Keffeler et al. (2021b). These enzymes allow *E*. *faecalis* to utilize poly-β1,4-linked N-acetylglucosamine, found in chitin, as carbon source. NAG sugar intake is then mediated by NagE and the Mpt glucose/mannose permease complex (MptBACD; Keffeler et al., 2021b).

HylA was identified as a MSCRAMM, thanks to its ligand-binding site including an Ig-like domain (Sillanpaa et al., 2004, 2009) and is considered as a virulence factor with adhesion properties (Nallapareddy et al., 2005). A previous study showed that a MSCRAMM of *E*. *faecalis* named EfbA can play an important role in maintenance through biofilm formation, in addition to its role in fibronectin adhesion and aortic valve colonization, in rat model (Singh et al., 2015). In the case of HylA, which also plays a role in biofilm formation, the protein does not fit this typical model, as its Ig-folded region is shorter than others and was suggested to have lower binding properties (Sillanpaa et al., 2009).

Proteins homologous to HylA have been found mainly in the genomes of the genera Enterococcus and Enterococcus_B (GTDB taxonomy), and in two forms: a short form with only the three lyase domains and a long form with additional domains in the N-and C-terminal regions (Figures 3, 6; Supplementary Figure 2C). The additional domains are involved in host-cell interactions, binding to cell wall associated proteins, or found in virulence factors that are produced by Gram-positive pathogens. The long form is found in *E*. *faecalis* and *E*. *hirae,* and is always associated to a RAT sequence. Our results show that these sequences were acquired by HGTs and that the presence of a RAT sequence places them under the control of NagY. Moreover, *E*. *faecalis* and *E*. *hirae* are both involved in enterococcal infections in humans (Agudelo Higuita and Huycke, 2014). Thus, this regulatory change and the presence of these additional domains confers novel properties to the HylA enzyme domain that may have contributed to the successful colonization of the gut by *E*. *faecalis*.

HylA of *E*. *faecalis* shares similarities with two polysaccharide lyases from pathogens like *Staphylococcus aureus* (HysA) and *Streptococcus pyogenes* (HylA), but the conservation of these sequences is only found for the lyase enzymatic domains (29% identity, 64% cover, and 28% identity, 46% cover, respectively). For similar coverage, these proteins are closer to the short Lyase_8 sequences of other *Enterococcaceae* (34 and 41% identity with protein from *E*. *cecorum* ATCC 43198, for example). Moreover, in *E*. *faecalis*, HylA is anchored to the envelope, contrarily to its hyaluronidase homologs in *S*. *aureus* and *S*. *pyogenes*. Many surface proteins are thought to be anchored to the cell wall of Gram-positive bacteria *via* their C-terminus. All surface proteins harboring an LPXTG sequence motif may therefore be cleaved and anchored by a universal mechanism (Navarre and Schneewind, 1994; Siegel et al., 2017; Bhat et al., 2021). We unexpectedly showed that HylA favors biofilm formation on GAG coating thanks to these domains, whereas hyaluronidases like HysA in *S*. *aureus* (Ibberson et al., 2016) are shown to be effective in dispersing biofilm, by cleaving glycosidic linkages of hyaluronic acid of the extracellular matrix. The fact that the biofilm dispersion phenotype is identical for Δ*nagY* and Δ*hylA* mutants supports that these genes belong to the same regulon.

In this report, we also observed that HylA is involved in *E*. *faecalis* colonization faculties in the *G*. *mellonella in vivo* model. This agrees with previous studies on Gram-positive pathogens, where HylA and its homologs were shown to be virulence factors (Hynes and Walton, 2000; Makris et al., 2004; Tsigrelis et al., 2006). Since hyaluronate is a major constituent of ECM, hyaluronidases are essential components to increase the permeability of the host environment, to weaken connective tissues and to allow the spread of pathogens from their initial site of infection (Hynes and Walton, 2000). The phylogenetic study showed that HylA was found only in the *E*. *faecalis* but with a very variable degree of sequence size conservation (Figure 7). Other HylA proteins like those of the pathogens *S*. *agalactiae* or *L*. *monocytogenes* have a domain organization very close to those of *E*. *faecalis* sequences, and the high sequence conservation between these distant species suggests recent HGTs. These observations suggest that the presence of a *hylA* gene would not be essential or would be counter-selected for *E*. *faecalis* strains in relation to their adaptative interactions with their host. Evidence from other Gram-positive pathogens shows that the adhesin family of MSCRAMM may serve as potential candidates for the development of novel immunotherapies (Rivas et al., 2004), opening interesting prospect for HylA in the future. We have established that NagY is able to regulate its own expression and the one of the HylA hyaluronidase, which is involved in the degradation of hyaluronic acid, a component of the host ECM, in biofilm formation and in pathogenicity. An interesting study performed on uropathogenic *E*. *coli* shown similar involvement of the PafR antiterminator in metabolism during colonization, with potential targets contributing to virulence traits like biofilm formation, adhesion or motility, and specifically expressed *in vivo* (Baum et al., 2014). In our Gram-positive bacterial model, this is the first evidence of an antiterminator regulon with direct target genes not only localized in the close genomic environment of the regulator gene. Consequently, the knowledge of NagY regulon may open up interesting perspective to decipher colonization mechanism of *E*. *faecalis* pathobiont.

## Data availability statement

The original contributions presented in the study are included in the article/Supplementary material; further inquiries can be directed to the corresponding authors.

## Author contributions

DS, MS, OL, AR, and CM designed the study and the research. YQ and GF conceived, designed, and performed the phylogenomic analyses. DS, MS, PL, NS, AB, OL, and CM performed the experiments. CM coordinates the project. DS, MS, PL, NS, AB, OL, YQ, GF, AR, and CM wrote the manuscript. All authors contributed to the article and approved the submitted version.

## Conflict of interest

The authors declare that the research was conducted in the absence of any commercial or financial relationships that could be construed as a potential conflict of interest.

## Publisher’s note

All claims expressed in this article are solely those of the authors and do not necessarily represent those of their affiliated organizations, or those of the publisher, the editors and the reviewers. Any product that may be evaluated in this article, or claim that may be made by its manufacturer, is not guaranteed or endorsed by the publisher.

## References

[ref1] Abdelhamed H. Lawrence M. L. Ramachandran R. Karsi A. (2019). Validation of predicted virulence factors in Listeria monocytogenes identified using comparative genomics. Toxins 11:E508. doi: 10.3390/toxins11090508, PMID: 31480280 PMC6783856

[ref2] Agudelo Higuita N. I. Huycke M. M. (2014). “Enterococcal disease, epidemiology, and implications for treatment” in Enterococci: From Commensals to Leading Causes of Drug Resistant Infection. eds. Gilmore M. S. Clewell D. B. Ike Y. Shankar N. (Boston: Massachusetts Eye and Ear Infirmary) 24649504

[ref3] Almagro Armenteros J. J. Tsirigos K. D. Sønderby C. K. Petersen T. N. Winther O. Brunak S.. (2019). SignalP 5.0 improves signal peptide predictions using deep neural networks. Nat. Biotechnol. 37, 420–423. doi: 10.1038/s41587-019-0036-z, PMID: 30778233

[ref4] Amster-Choder O. (2005). The bgl sensory system: a transmembrane signaling pathway controlling transcriptional antitermination. Curr. Opin. Microbiol. 8, 127–134. doi: 10.1016/j.mib.2005.02.014, PMID: 15802242

[ref5] Archambaud C. Derré-Bobillot A. Lapaque N. Rigottier-Gois L. Serror P. (2019). Intestinal translocation of enterococci requires a threshold level of enterococcal overgrowth in the lumen. Sci. Rep. 9:8926. doi: 10.1038/s41598-019-45441-3, PMID: 31222056 PMC6586816

[ref6] Arnaud M. Débarbouillé M. Rapoport G. Saier M. H. Reizer J. (1996). In vitro reconstitution of transcriptional antitermination by the SacT and SacY proteins of Bacillus subtilis. J. Biol. Chem. 271, 18966–18972. doi: 10.1074/jbc.271.31.18966, PMID: 8702561

[ref7] Aymerich S. Steinmetz M. (1992). Specificity determinants and structural features in the RNA target of the bacterial antiterminator proteins of the BglG/SacY family. Proc. Natl. Acad. Sci. U. S. A. 89, 10410–10414. doi: 10.1073/pnas.89.21.10410, PMID: 1279678 PMC50348

[ref8] Bailey T. L. Elkan C. (1994). Fitting a mixture model by expectation maximization to discover motifs in biopolymers. Proc. Int. Conf. Intell. Syst. Mol. Biol. 2, 28–36. 7584402

[ref9] Barnes A. M. T. Frank K. L. Dunny G. M. (2021). Enterococcal endocarditis: hiding in plain sight. Front. Cell. Infect. Microbiol. 11:722482. doi: 10.3389/fcimb.2021.722482, PMID: 34527603 PMC8435889

[ref10] Baum M. Watad M. Smith S. N. Alteri C. J. Gordon N. Rosenshine I.. (2014). PafR, a novel transcription regulator, is important for pathogenesis in uropathogenic Escherichia coli. Infect. Immun. 82, 4241–4252. doi: 10.1128/IAI.00086-14, PMID: 25069986 PMC4187875

[ref11] Benachour A. Ladjouzi R. Le Jeune A. Hébert L. Thorpe S. Courtin P.. (2012). The lysozyme-induced peptidoglycan N-acetylglucosamine deacetylase PgdA (EF1843) is required for Enterococcus faecalis virulence. J. Bacteriol. 194, 6066–6073. doi: 10.1128/JB.00981-12, PMID: 22961856 PMC3486378

[ref12] Bhat A. H. Nguyen M. T. Das A. Ton-That H. (2021). Anchoring surface proteins to the bacterial cell wall by sortase enzymes: how it started and what we know now. Curr. Opin. Microbiol. 60, 73–79. doi: 10.1016/j.mib.2021.01.013, PMID: 33611145 PMC7990056

[ref13] Capella-Gutiérrez S. Silla-Martínez J. M. Gabaldón T. (2009). trimAl: a tool for automated alignment trimming in large-scale phylogenetic analyses. Bioinforma. Oxf. Engl. 25, 1972–1973. doi: 10.1093/bioinformatics/btp348, PMID: 19505945 PMC2712344

[ref14] Chang D.-E. Smalley D. J. Tucker D. L. Leatham M. P. Norris W. E. Stevenson S. J.. (2004). Carbon nutrition of Escherichia coli in the mouse intestine. Proc. Natl. Acad. Sci. 101, 7427–7432. doi: 10.1073/pnas.0307888101, PMID: 15123798 PMC409935

[ref15] Clerte C. Declerck N. Margeat E. (2013). Competitive folding of anti-terminator/terminator hairpins monitored by single molecule FRET. Nucleic Acids Res. 41, 2632–2643. doi: 10.1093/nar/gks1315, PMID: 23303779 PMC3575810

[ref16] Darriba D. Posada D. Kozlov A. M. Stamatakis A. Morel B. Flouri T. (2020). ModelTest-NG: a new and scalable tool for the selection of DNA and protein evolutionary models. Mol. Biol. Evol. 37, 291–294. doi: 10.1093/molbev/msz189, PMID: 31432070 PMC6984357

[ref17] Deutscher J. Francke C. Postma P. W. (2006). How phosphotransferase system-related protein phosphorylation regulates carbohydrate metabolism in bacteria. Microbiol. Mol. Biol. Rev. 70, 939–1031. doi: 10.1128/MMBR.00024-06, PMID: 17158705 PMC1698508

[ref18] Eddy S. R. (2011). Accelerated profile HMM searches. PLoS Comput. Biol. 7:e1002195. doi: 10.1371/journal.pcbi.1002195, PMID: 22039361 PMC3197634

[ref19] Faron M. L. Ledeboer N. A. Buchan B. W. (2016). Resistance mechanisms, epidemiology, and approaches to screening for vancomycin-resistant Enterococcus in the health care setting. J. Clin. Microbiol. 54, 2436–2447. doi: 10.1128/JCM.00211-16, PMID: 27147728 PMC5035425

[ref20] Fernández-Hidalgo N. Escolà-Vergé L. Pericàs J. M. (2020). Enterococcus faecalis endocarditis: what’s next? Future Microbiol. 15, 349–364. doi: 10.2217/fmb-2019-0247, PMID: 32286105

[ref21] Fiore E. Van Tyne D. Gilmore M. S. (2019). Pathogenicity of enterococci. Microbiol. Spectr. 7, 1–23. doi: 10.1128/microbiolspec.GPP3-0053-2018, PMID: 31298205 PMC6629438

[ref22] Galinier A. Deutscher J. (2017). Sophisticated regulation of transcriptional factors by the bacterial phosphoenolpyruvate: sugar phosphotransferase system. J. Mol. Biol. 429, 773–789. doi: 10.1016/j.jmb.2017.02.006, PMID: 28202392

[ref23] García-Solache M. Rice L. B. (2019). The enterococcus: a model of adaptability to its environment. Clin. Microbiol. Rev. 32, 1–28. doi: 10.1128/CMR.00058-18, PMID: 30700430 PMC6431128

[ref24] Gordon N. Rosenblum R. Nussbaum-Shochat A. Eliahoo E. Amster-Choder O. (2015). A search for ribonucleic antiterminator sites in bacterial genomes: not only antitermination? J. Mol. Microbiol. Biotechnol. 25, 143–153. doi: 10.1159/000375263, PMID: 26159075

[ref25] Görke B. (2003). Regulation of the Escherichia coli antiterminator protein BglG by phosphorylation at multiple sites and evidence for transfer of phosphoryl groups between monomers. J. Biol. Chem. 278, 46219–46229. doi: 10.1074/jbc.M308002200, PMID: 12963714

[ref26] Görke B. Stülke J. (2008). Carbon catabolite repression in bacteria: many ways to make the most out of nutrients. Nat. Rev. Microbiol. 6, 613–624. doi: 10.1038/nrmicro1932, PMID: 18628769

[ref27] Habib G. Erba P. A. Iung B. Donal E. Cosyns B. Laroche C.. (2019). Clinical presentation, aetiology and outcome of infective endocarditis. Results of the ESC-EORP EURO-ENDO (European infective endocarditis) registry: a prospective cohort study. Eur. Heart J. 40, 3222–3232. doi: 10.1093/eurheartj/ehz620, PMID: 31504413

[ref28] Hendrickx A. P. A. Willems R. J. L. Bonten M. J. M. van Schaik W. (2009). LPxTG surface proteins of enterococci. Trends Microbiol. 17, 423–430. doi: 10.1016/j.tim.2009.06.004, PMID: 19726195

[ref29] Huang F. Spangler J. R. Huang A. Y. (2017). In vivo cloning of up to 16 kb plasmids in E. coli is as simple as PCR. PLoS One 12:e0183974. doi: 10.1371/journal.pone.0183974, PMID: 28837659 PMC5570364

[ref30] Hynes W. L. Walton S. L. (2000). Hyaluronidases of gram-positive bacteria. FEMS Microbiol. Lett. 183, 201–207. doi: 10.1111/j.1574-6968.2000.tb08958.x 10675584

[ref31] Ibberson C. B. Parlet C. P. Kwiecinski J. Crosby H. A. Meyerholz D. K. Horswill A. R. (2016). Hyaluronan modulation impacts Staphylococcus aureus biofilm infection. Infect. Immun. 84, 1917–1929. doi: 10.1128/IAI.01418-15, PMID: 27068096 PMC4907140

[ref32] Innocenti N. Golumbeanu M. Fouquier d’Hérouël A. Lacoux C. Bonnin R. A. Kennedy S. P.. (2015). Whole-genome mapping of 5’ RNA ends in bacteria by tagged sequencing: a comprehensive view in Enterococcus faecalis. RNA 21, 1018–1030. doi: 10.1261/rna.048470.114, PMID: 25737579 PMC4408782

[ref33] Kanehisa M. Furumichi M. Tanabe M. Sato Y. Morishima K. (2017). KEGG: new perspectives on genomes, pathways, diseases and drugs. Nucleic Acids Res. 45, D353–D361. doi: 10.1093/nar/gkw1092, PMID: 27899662 PMC5210567

[ref34] Kao P. H. N. Kline K. A. (2019). Dr. Jekyll and Mr. Hide: how Enterococcus faecalis subverts the host immune response to cause infection. J. Mol. Biol. 431, 2932–2945. doi: 10.1016/j.jmb.2019.05.030, PMID: 31132360

[ref35] Katoh K. Standley D. M. (2013). MAFFT multiple sequence alignment software version 7: improvements in performance and usability. Mol. Biol. Evol. 30, 772–780. doi: 10.1093/molbev/mst010, PMID: 23329690 PMC3603318

[ref36] Kawai K. Kamochi R. Oiki S. Murata K. Hashimoto W. (2018). Probiotics in human gut microbiota can degrade host glycosaminoglycans. Sci. Rep. 8:10674. doi: 10.1038/s41598-018-28886-w, PMID: 30006634 PMC6045597

[ref37] Keffeler E. C. Iyer V. S. Parthasarathy S. Ramsey M. M. Gorman M. J. Barke T. L.. (2021a). Influence of the alternative sigma factor RpoN on global gene expression and carbon catabolism in Enterococcus faecalis V583. MBio 12, e00380–e00321. doi: 10.1128/mBio.00380-21 34006651 PMC8262876

[ref38] Keffeler E. C. Parthasarathy S. Abdullahi Z. H. Hancock L. E. (2021b). Metabolism of poly-β1,4-N-acetylglucosamine substrates and importation of N-acetylglucosamine and glucosamine by Enterococcus faecalis. J. Bacteriol. 203:e0037121. doi: 10.1128/JB.00371-21, PMID: 34424034 PMC8508097

[ref39] Kelly G. Prasannan S. Daniell S. Fleming K. Frankel G. Dougan G.. (1999). Structure of the cell-adhesion fragment of intimin from enteropathogenic Escherichia coli. Nat. Struct. Biol. 6, 313–318. doi: 10.1038/7545, PMID: 10201396

[ref40] Kundig W. Ghosh S. Roseman S. (1964). Phosphate bound to histidine in a protein as an intermediate in a novel phosphotransferase system. Proc. Natl. Acad. Sci. 52, 1067–1074. doi: 10.1073/pnas.52.4.1067, PMID: 14224387 PMC300396

[ref41] La Carbona S. Sauvageot N. Giard J.-C. Benachour A. Posteraro B. Auffray Y.. (2007). Comparative study of the physiological roles of three peroxidases (NADH peroxidase, alkyl hydroperoxide reductase and thiol peroxidase) in oxidative stress response, survival inside macrophages and virulence of Enterococcus faecalis. Mol. Microbiol. 66, 1148–1163. doi: 10.1111/j.1365-2958.2007.05987.x, PMID: 17971082

[ref42] Lebreton F. Manson A. L. Saavedra J. T. Straub T. J. Earl A. M. Gilmore M. S. (2017). Tracing the enterococci from paleozoic origins to the hospital. Cells 169, 849–861.e13. doi: 10.1016/j.cell.2017.04.027, PMID: 28502769 PMC5499534

[ref43] Letunic I. Bork P. (2019). Interactive tree of life (iTOL) v4: recent updates and new developments. Nucleic Acids Res. 47, W256–W259. doi: 10.1093/nar/gkz239, PMID: 30931475 PMC6602468

[ref44] Lombard V. Golaconda Ramulu H. Drula E. Coutinho P. M. Henrissat B. (2014). The carbohydrate-active enzymes database (CAZy) in 2013. Nucleic Acids Res. 42, D490–D495. doi: 10.1093/nar/gkt1178, PMID: 24270786 PMC3965031

[ref45] Lorenz R. Bernhart S. H. Hönerzu Siederdissen C. Tafer H. Flamm C. Stadler P. F.. (2011). ViennaRNA package 2.0. Algorithms. Mol. Biol. 6:26. doi: 10.1186/1748-7188-6-26, PMID: 22115189 PMC3319429

[ref46] Ludwig W. Schleifer K.-H. Whitman W. B. (2009). “Revised road map to the phylum Firmicutes” in Bergey’s Manual of Systematic Bacteriology, vol. 13 (New York, NY: Springer).

[ref47] Makris G. Wright J. D. Ingham E. Holland K. T. (2004). The hyaluronate lyase of Staphylococcus aureus—a virulence factor? Microbiology 150, 2005–2013. doi: 10.1099/mic.0.26942-0, PMID: 15184586

[ref48] Mao F. Dam P. Chou J. Olman V. Xu Y. (2009). DOOR: a database for prokaryotic operons. Nucleic Acids Res. 37, D459–D463. doi: 10.1093/nar/gkn757, PMID: 18988623 PMC2686520

[ref49] Michaux C. Hansen E. E. Jenniches L. Gerovac M. Barquist L. Vogel J. (2020). Single-nucleotide RNA maps for the two major nosocomial pathogens enterococcus faecalis and Enterococcus faecium. Front. Cell. Infect. Microbiol. 10:600325. doi: 10.3389/fcimb.2020.600325, PMID: 33324581 PMC7724050

[ref50] Minh B. Q. Schmidt H. A. Chernomor O. Schrempf D. Woodhams M. D. von Haeseler A.. (2020). IQ-TREE 2: new models and efficient methods for phylogenetic inference in the genomic era. Mol. Biol. Evol. 37, 1530–1534. doi: 10.1093/molbev/msaa015, PMID: 32011700 PMC7182206

[ref51] Morel B. Kozlov A. M. Stamatakis A. Szöllősi G. J. (2020). GeneRax: a tool for species-tree-aware maximum likelihood-based gene family tree inference under gene duplication, transfer, and loss. Mol. Biol. Evol. 37, 2763–2774. doi: 10.1093/molbev/msaa141, PMID: 32502238 PMC8312565

[ref52] Muller C. Cacaci M. Sauvageot N. Sanguinetti M. Rattei T. Eder T.. (2015). The intraperitoneal transcriptome of the opportunistic pathogen Enterococcus faecalis in mice. PLoS One 10:e0126143. doi: 10.1371/journal.pone.0126143, PMID: 25978463 PMC4433114

[ref53] Nallapareddy S. R. Wenxiang H. Weinstock G. M. Murray B. E. (2005). Molecular characterization of a widespread, pathogenic, and antibiotic resistance-receptive Enterococcus faecalis lineage and dissemination of its putative pathogenicity island. J. Bacteriol. 187, 5709–5718. doi: 10.1128/JB.187.16.5709-5718.2005, PMID: 16077117 PMC1196071

[ref54] Navarre W. W. Schneewind O. (1994). Proteolytic cleavage and cell wall anchoring at the LPXTG motif of surface proteins in gram-positive bacteria. Mol. Microbiol. 14, 115–121. doi: 10.1111/j.1365-2958.1994.tb01271.x, PMID: 7830549

[ref55] Nawrocki E. P. Eddy S. R. (2013). Infernal 1.1: 100-fold faster RNA homology searches. Bioinformatics 29, 2933–2935. doi: 10.1093/bioinformatics/btt509, PMID: 24008419 PMC3810854

[ref56] Ondov B. D. Treangen T. J. Melsted P. Mallonee A. B. Bergman N. H. Koren S.. (2016). Mash: fast genome and metagenome distance estimation using MinHash. Genome Biol. 17:132. doi: 10.1186/s13059-016-0997-x, PMID: 27323842 PMC4915045

[ref57] Parks D. H. Chuvochina M. Rinke C. Mussig A. J. Chaumeil P.-A. Hugenholtz P. (2022). GTDB: an ongoing census of bacterial and archaeal diversity through a phylogenetically consistent, rank normalized and complete genome-based taxonomy. Nucleic Acids Res. 50, D785–D794. doi: 10.1093/nar/gkab776, PMID: 34520557 PMC8728215

[ref58] Paulsen I. T. Banerjei L. Myers G. S. A. Nelson K. E. Seshadri R. Read T. D.. (2003). Role of mobile DNA in the evolution of vancomycin-resistant Enterococcus faecalis. Science 299, 2071–2074. doi: 10.1126/science.1080613, PMID: 12663927

[ref59] Prasad I. Schaefler S. (1974). Regulation of the β-glucoside system in Escherchia coli K-12. J. Bacteriol. 120, 638–650. doi: 10.1128/jb.120.2.638-650.1974, PMID: 4616943 PMC245822

[ref60] Ramsey M. Hartke A. Huycke M. (2014). “The physiology and metabolism of enterococci” in Enterococci: from commensals to leading causes of drug resistant infection. eds. Gilmore M. S. Clewell D. B. Ike Y. Shankar N. (Boston: Massachusetts Eye and Ear Infirmary) 24649510

[ref61] Rivas J. M. Speziale P. Patti J. M. Höök M. (2004). MSCRAMM--targeted vaccines and immunotherapy for staphylococcal infection. Curr. Opin. Drug Discov. Devel. 7, 223–227. 15603256

[ref62] Salze M. Giard J.-C. Riboulet-Bisson E. Hain T. Rincé A. Muller C. (2020a). Identification of the general stress stimulon related to colonization in Enterococcus faecalis. Arch. Microbiol. 202, 233–246. doi: 10.1007/s00203-019-01735-8, PMID: 31599337

[ref63] Salze M. Muller C. Bernay B. Hartke A. Clamens T. Lesouhaitier O.. (2020b). Study of key RNA metabolism proteins in Enterococcus faecalis. RNA Biol. 17, 794–804. doi: 10.1080/15476286.2020.1728103, PMID: 32070211 PMC7549701

[ref64] Seemann T. (2014). Prokka: rapid prokaryotic genome annotation. Bioinforma. Oxf. Engl. 30, 2068–2069. doi: 10.1093/bioinformatics/btu153, PMID: 24642063

[ref65] Shioya K. Michaux C. Kuenne C. Hain T. Verneuil N. Budin-Verneuil A.. (2011). Genome-wide identification of small RNAs in the opportunistic pathogen Enterococcus faecalis V583. PLoS One 6:e23948. doi: 10.1371/journal.pone.0023948, PMID: 21912655 PMC3166299

[ref66] Siegel S. D. Reardon M. E. Ton-That H. (2017). Anchoring of LPXTG-like proteins to the gram-positive cell wall envelope. Curr. Top. Microbiol. Immunol. 404, 159–175. doi: 10.1007/82_2016_8, PMID: 27097813

[ref67] Sillanpaa J. Nallapareddy S. R. Houston J. Ganesh V. K. Bourgogne A. Singh K. V.. (2009). A family of fibrinogen-binding MSCRAMMs from Enterococcus faecalis. Microbiology 155, 2390–2400. doi: 10.1099/mic.0.027821-0, PMID: 19389755 PMC2739004

[ref68] Sillanpaa J. Xu Y. Nallapareddy S. R. Murray B. E. Hook M. (2004). A family of putative MSCRAMMs from Enterococcus faecalis. Microbiology 150, 2069–2078. doi: 10.1099/mic.0.27074-0, PMID: 15256550

[ref69] Singh K. V. La Rosa S. L. Somarajan S. R. Roh J. H. Murray B. E. (2015). The fibronectin-binding protein EfbA contributes to pathogenesis and protects against infective endocarditis caused by Enterococcus faecalis. Infect. Immun. 83, 4487–4494. doi: 10.1128/IAI.00884-15, PMID: 26351286 PMC4645374

[ref70] Steinegger M. Söding J. (2017). MMseqs2 enables sensitive protein sequence searching for the analysis of massive data sets. Nat. Biotechnol. 35, 1026–1028. doi: 10.1038/nbt.3988, PMID: 29035372

[ref71] Steinmetz M. Aymerich S. Le Coq D. Gonzy-Tréboul G. (1988). “Levansucrase induction by sucrose in Bacillus subtilis involves an antiterminator. Homology with the Escherichia coli bgl operon” in Genetics and biotechnology of bacilli. eds. Ganesan A. T. Hoch J. A. (New York: Academic Press Book), 11–16.

[ref72] Stern R. Jedrzejas M. J. (2006). The hyaluronidases: their genomics, structures, and mechanisms of action. Chem. Rev. 106, 818–839. doi: 10.1021/cr050247k, PMID: 16522010 PMC2547145

[ref73] Stülke J. (2002). Control of transcription termination in bacteria by RNA-binding proteins that modulate RNA structures. Arch. Microbiol. 177, 433–440. doi: 10.1007/s00203-002-0407-5 12029388

[ref74] Stülke J. Arnaud M. Rapoport G. Martin-Verstraete I. (1998). PRD? A protein domain involved in PTS-dependent induction and carbon catabolite repression of catabolic operons in bacteria. Mol. Microbiol. 28, 865–874. doi: 10.1046/j.1365-2958.1998.00839.x, PMID: 9663674

[ref75] Suárez C. A. Blancato V. S. Poncet S. Deutscher J. Magni C. (2011). CcpA represses the expression of the divergent cit operons of Enterococcus faecalis through multiple cre sites. BMC Microbiol. 11:227. doi: 10.1186/1471-2180-11-227, PMID: 21989394 PMC3198936

[ref76] Tettelin H. Masignani V. Cieslewicz M. J. Donati C. Medini D. Ward N. L.. (2005). Genome analysis of multiple pathogenic isolates of Streptococcus agalactiae: implications for the microbial pan-genome. Proc. Natl. Acad. Sci. U. S. A. 102, 13950–13955. doi: 10.1073/pnas.0506758102, PMID: 16172379 PMC1216834

[ref77] Theocharis A. D. Skandalis S. S. Gialeli C. Karamanos N. K. (2016). Extracellular matrix structure. Adv. Drug Deliv. Rev. 97, 4–27. doi: 10.1016/j.addr.2015.11.001 26562801

[ref78] Thurlow L. R. Thomas V. C. Hancock L. E. (2009). Capsular polysaccharide production in Enterococcus faecalis and contribution of CpsF to capsule serospecificity. J. Bacteriol. 191, 6203–6210. doi: 10.1128/JB.00592-09, PMID: 19684130 PMC2753019

[ref79] Tonkin-Hill G. MacAlasdair N. Ruis C. Weimann A. Horesh G. Lees J. A.. (2020). Producing polished prokaryotic pangenomes with the Panaroo pipeline. Genome Biol. 21:180. doi: 10.1186/s13059-020-02090-4, PMID: 32698896 PMC7376924

[ref80] Tortosa P. Declerck N. Dutartre H. Lindner C. Deutscher J. Le Coq D. (2001). Sites of positive and negative regulation in the Bacillus subtilis antiterminators LicT and SacY. Mol. Microbiol. 41, 1381–1393. doi: 10.1046/j.1365-2958.2001.02608.x, PMID: 11580842

[ref81] Tortosa P. Le Coq D. (1995). A ribonucleic antiterminator sequence (RAT) and a distant palindrome are both involved in sucrose induction of the Bacillus subtilis sacXY regulatory operon. Microbiol. Read. Engl. 141, 2921–2927. doi: 10.1099/13500872-141-11-2921 8535520

[ref82] Tsigrelis C. Singh K. V. Coutinho T. D. Murray B. E. Baddour L. M. (2006). Vancomycin-resistant Enterococcus faecalis endocarditis: linezolid failure and strain characterization of virulence factors. J. Clin. Microbiol. 45, 631–635. doi: 10.1128/jcm.02188-06, PMID: 17182759 PMC1829077

[ref83] Ubeda C. Taur Y. Jenq R. R. Equinda M. J. Son T. Samstein M.. (2010). Vancomycin-resistant Enterococcus domination of intestinal microbiota is enabled by antibiotic treatment in mice and precedes bloodstream invasion in humans. J. Clin. Invest. 120, 4332–4341. doi: 10.1172/JCI43918, PMID: 21099116 PMC2993598

[ref84] Will S. Joshi T. Hofacker I. L. Stadler P. F. Backofen R. (2012). LocARNA-P: accurate boundary prediction and improved detection of structural RNAs. RNA 18, 900–914. doi: 10.1261/rna.029041.111, PMID: 22450757 PMC3334699

[ref85] Zhou Z. Charlesworth J. Achtman M. (2020). Accurate reconstruction of bacterial pan-and core genomes with PEPPAN. Genome Res. 30, 1667–1679. doi: 10.1101/gr.260828.120, PMID: 33055096 PMC7605250

