# Supplementary material for: The NagY regulator: A member of the BglG/SacY antiterminator family conserved in Enterococcus faecalis and involved in virulence
Source: Front Microbiol. 2023 Feb 17;13:1070116. doi: 10.3389/fmicb.2022.1070116 (PMC9981650; doi:10.3389/fmicb.2022.1070116)
Supplement: Supplementary file 1 [file Data_Sheet_1.pdf]

Supplementary Material and Methods

1 Supplementary Methods

Growth experiments

E. faecalis overnight cultures in GM17 were washed with carbon depleted medium cdM17 (La Carbona et al., 2007) and the OD_{600} was adjusted to 0.02 in cdM17. The cultures were then performed in microplate, with media supplemented with 0.5% glucose, N-acetylglucosamine (NAG), or hyaluronic acid, chondroitin sulfate or heparin sodium sonicated 15 min, and incubated at 37°C for 24 h in anaerobic conditions. The OD was measured using a Model 680 Microplate reader (Bio-Rad, Hercules, Californie, USA).

RT-PCR

Reverse Transcription for RT-PCR assay to verify *nagY* and *nagE* cotranscription was performed using QuantiTect Reverse Transcription kit (Qiagen) and GoTaq DNA Polymerase (Promega) with ef1515RTL and ef1516RTR oligonucleotides (Supplementary Table 2).

RACE-PCR

5'RACE-PCR were performed using the 5'/3' RACE kit, 2nd generation (Roche), with poly-A/C/G tailing. PCR were performed using Phusion High-Fidelity DNA Polymerase (ThermoFisher), with primers ef1515R, ef1515_SP2, ef1515_SP3 and ef3023_SP1, ef3023_SP2, ef3023_SP3 for *nagY* and *hylA*, respectively (Supplementary table 2). PCR products were purified when necessary, using the NucleoSpin Gel and PCR Clean-up kit (Macherey-Nagel) and plasmid extractions were achieved with the NucleoSpin Plasmid kit (Macherey-Nagel) according to the manufacturer's recommendations.

Validation of *E. faecalis* mutant strains by whole genome sequencing

Chromosomal DNA of V19, Δ*nagY*, Δ*5'nagY* and Δ*hylA* strains was extracted with the Nucleospin Microbial DNA (Macherey-Nagel) according to the manufacturer's recommendations. DNA quantities were measured using a Qubit 4.0 fluorometer (Thermo Fisher Scientific, Waltham, MA, USA) and integrity was checked by electrophoresis. Genomic DNA was sequenced using a MiSeq instrument (Illumina, Inc., San Diego, California) with paired-end and barcode strategy according to the Illumina DNA prep and the Nextera DNA CD Indexes kits (Illumina, Inc., San Diego, California). The libraries were normalized, pooled, then loaded onto the Miseq Reagent Kit V2 (300 cycles). The quality of the reads was checked using FastQC v.0.11.9 (Andrews, 2010). Sequence analysis was performed using the "*CLC Genomics Workbench*" software (version 12.0.2) (CLC Bio, Walthan, MA), with the variant detection tool by comparing with V19 WT sequence and the reference genome of *E. faecalis* V583 (NCBI GenBank: NC_004668). All programs were used with default parameters.

The sequencing resulted in an output of paired-end read sets containing 3,353,996, 3,254,040, 3,712,254 and 2,882,474 reads for the V19 Δ*nagY*, Δ*hylA*, and Δ*5'nagY* mutant strains, respectively. Differences between WT and mutant strains are listed and discussed in Supplementary Table 3.

Data availability

Genomes sequences have been deposited in the GenBank database, with the BioProject number PRJNA912900. The Sequence Read Archive (SRA) submission is available under the accession number SRR22763427, SRR22763428, SRR22763429, SRR22763430.

Phylogenomic analyses

Pan genomes. To analyze the pan-genomes, we used two software packages depending on the observed evolutionary distance between genomes. For *Enterococcus* species, *PEPPAN* was chosen because it can reliably construct pan-genomes from genetically diverse bacterial genomes up to an entire genus (Zhou et al., 2020). For the *E. faecalis* strain pan-genome, we selected *Panaroo* (version 1.2.10) which is better suited to address the analysis of strains of the same species and has the advantage of re-analyzing genome sequences to propose gene annotation corrections (Tonkin-Hill et al., 2020). The match_identity parameter from *PEPPAN* controls the minimal identity of an alignment to be considered in the pan-genome construction. Several values were tested and two thresholds where selected: 0.65 for inferring the species tree and a more relaxed value (0.5) for analyzing gene families. *Panaroo* was used with the most conservative mode and with the option to generate alignments of sequences belonging to the core genome with the *mafft* software (parameters: --clean-mode strict --merge_paralogs --alignment core --aligner mafft --core_threshold 0.95).

***Enterococcaceae* species tree**. In order to obtain a rooting of the *Enterococcus* tree, we first used an outgroup in addition to the *Enterococcus* genomes using the alignment available in GTDB. Then, we recomputed a tree with only *Enterococcus* sequences and an alignment with a larger number of informative sites. In this way we eliminate the biases induced by the presence of the most divergent sequences and increase the phylogenetic signal to obtain a better estimate of the *Enterococcus* species tree. This tree is then rooted on the node corresponding to the last common *Enterococcus* ancestor identified on the first tree. In practice, we used the 120 single copy markers identified as reliable for phylogenetic inference. To the 81 *Enterococcus* strains, seven representative *Lactobacillales* species obtained from GTDB were included in the sample to act as an outgroup among which one genome from both *Vagococcaceae* and *Carnobacteriaceae* families previously used as outgroup (Lebreton et al., 2017). The tree was calculated with *IQ-TREE* (version 2.2.0) (Minh et al., 2020), with parameters: *-nt AUTO --ufboot 1000 --bnni --alrt 1000* with the LG+F+I+R6 model, and the branch support values were estimated with ultrafast bootstrap approximation (Hoang et al., 2018) and the SH-like approximate likelihood ratio test (Guindon et al., 2010). In Supplementary Figure 1, the subtree composed of two *Enterococcus sp.* from Marseille-P2817 strains (GCA_900104595.1 and GCA_005405245.1) is the closest of the *Lactobacillales* group (bootstrap support 100/100) and appears as the outgroup of the *Enterococcus* strains (bootstrap support 99.8/100). Therefore, these two sequences were used to root the *Enterococcus* species tree in Figure 3.

2 Supplementary Figures and Tables

Supplementary Table 1. Strains and plasmids used in this study.

	Name	Characteristics	Reference
Strain	*E. faecalis* V19	Plasmid-cured strain derived from the V583	Paulsen et al., 2003
	E. faecalis V19 Δ*nagY*	*E. faecalis* V19 deleted for the *nagY* gene	This study
	E. faecalis V19 Δ5'*nagY*	*E. faecalis* V19 deleted for the 5' UTR of the *nagY* gene	This study
	E. faecalis V19 Δ*hylA*	*E. faecalis* V19 deleted for the *hylA* gene	This study
	E. coli TOP10		ThermoFisher
	E. coli NEB-5α		New England BioLabs
	E. coli M15 pRep4	*E. coli* M15 containing the plasmid pRep4, KanR	Qiagen
Plasmid	pTOPO	KanR	ThermoFisher
	pQE70	AmpR	Qiagen
	pLT06	CmR	Thurlow et al., 2009

Supplementary Table 2. Primers used in this study.

Name	Sequence	Use
ef1515_SP2	TATTAAACCCGACGCCTTTAC	RACE-PCR
ef1515_SP3	TTCTCCTGTCCTTCGTCAAG	RACE-PCR
ef1515L	AGTAGCTGTCGGTAAAGGCG	qPCR
ef1515R	AGTCCTTCTGGCTCCATCAC	qPCR, RACE-PCR
ef1515RTL	GAAAGCTATGAAATCTCAAAA	RT-PCR
ef1516RTR	CATTCATTAGAAAAGTTGTG	RT-PCR
ef1516L	ATGTGCACGTCCTGAGAAGA	qPCR
ef1516R	GCCAGCTGTCCAATGCATAA	qPCR
GyrAL	GATGGGGAAATCAGGGATTC	qPCR
GyrAR	TCTTTTCCATTCGGCATTTC	qPCR
Ef3023_SP1	AATCAACAATTAACCATTGTTCA	RACE-PCR
Ef3023_SP2	ACAAATTGAGGGTATTTCCAAT	RACE-PCR
Ef3023_SP3	TAAGCATGAAAGCAACAACGA	RACE-PCR
pLT06_1_bis	GAATTCGAGCTCGGTACC	Mutant construction
pLT06_2	CTGCAGGTCGATAAACCC	Mutant construction
ef1515_1	ACGATCCCCGGGTACCGAGCTCGAATTCAAAAGTCTATTCCTTACGTT	Mutant construction
ef1515_2	TCAATGTGAATCGATGTCAAGAACAAGTACAGCATTT	Mutant construction
ef1515_3	TGTACTTGTTCTTGACATCGATTCACATTGAACGA	Mutant construction
ef1515_4	GTTCGCTGGGTTTATCGACCTGCAGTTCAAACTTAAGAAGAAGTCTACAA	Mutant construction
ef1515_6	CCAACATCATTGGATGATTCG	Mutant construction
ef3023_1	ATCCCCGGGTACCGAGCTCGAATTCTAGGTGAAAATTAAACTAGTAACAG	Mutant construction
ef3023_2	CGATAACTAGAAGACATGAAAGCAACAACGACTATTA	Mutant construction
ef3023_3	CGTTGTTGCTTTCATGTCTTCTAGTTATCGCCAGT	Mutant construction
ef3023_4	ATGGTTCGCTGGGTTTATCGACCTGCAGCTTTTAATGTGTCGTTATCTGA	Mutant construction
ef3023_5	GTGGAAAGTAGCCAAACTTC	Mutant construction
ef3023_6	TGGAATTGTGGGCTATAGC	Mutant construction
topo_FP2	GAATTCCAGCACACTGG	pTOPO cloning
topo_RP2	CTATAGTGAGTCGTATTACA	pTOPO cloning
topo65_FP1	TAATACGACTCACTATAGTATCATAATAAAGGAGGTGAAAT	pTOPO cloning
topo65_RP1	AGTGTGCTGGAATTCAATTTATAATTTGTGTTATACTAAG	pTOPO cloning
topo85_FP1	TAATACGACTCACTATAGTTAACAAATGAATAGCGTTTTC	pTOPO cloning
topo85_RP1	AGTGTGCTGGAATTCATGACCCTCTTCTGCAGAA	pTOPO cloning
topo5'3023_FP1	TAATACGACTCACTATAGCGTAAACATGTATAATATTTCATG	pTOPO cloning
topo5'3023_RP1	AGTGTGCTGGAATTCCAATCCGACTTTTCCAAACAC	pTOPO cloning
ef1515_pQE70_*SphI*	TACGCATGCGAATTAAAAAGGTGCTAAATCAAAA	pQE70 cloning
ef1515_pQE70_*BglII*	ATGAGATCTTTTGGTCATTCTTAATCGTTCA	pQE70 cloning

Supplementary Table 3. Results of the variant detection analysis of the genomes of mutants constructed in this study. SNV : Single Nucleotide Variant.

Mutant	Genome Position	Location and annotation	Variant type	Nucleotide and amino-acid changes	Comments
Δ*nagY*	2920112	*ef3044*/EF_RS14425 *nagA* N-acetylglucosamine-6-phosphate deacetylase	SNV	TGT to TGC Cys to Cys	No amino-acid change
Δ*hylA*	3016608	*ef3144*/EF_RS14890 MurR/RpiR family transcriptionnal regulator	SNV	AAC to AAT Asn to Asn	No amino-acid change
	2835039	*ef2962*/EF_RS14050 LacI family transcriptional regulator	SNV	TCC to TCA Ser to Ser	No amino-acid change
Δ*5'nagY*	134299	Between *ef0129*/EF_RS00580 helix-turn-helix transcriptional regulator and *ef0130*/EF_RS00585 hypothetical protein	SNV	G to T	*ef0129* and *ef0130* and are not co-transcribed. Mutation is not located in 5' and 3'UTR of these genes.

Supplementary Table 4. ***Enterococcaceae* reference species used in this study**. .xlsx file.

Supplementary Table 5. ***Enterococcaceae* orthologous genes.** .xlsx file.

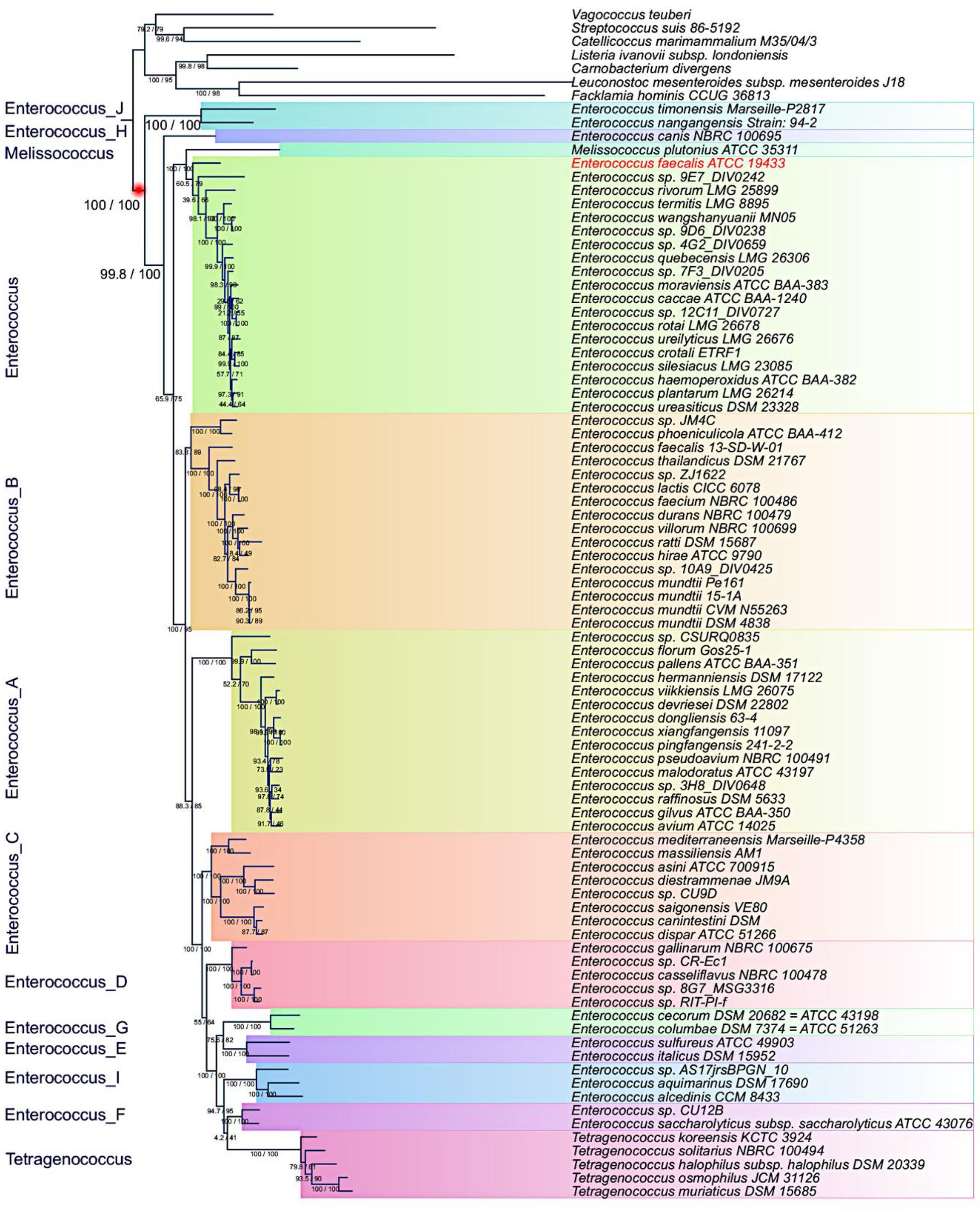

Supplementary Figure 1. *Enterococcaceae* species tree with *Lactobacillales* species as outgroup. The tree inferred with the 120 single copy markers (Parks et al., 2017) and rooted with seven representative *Lactobacillales* species obtained from GTDB. A red dot indicates the position of the last common ancestor of the *Enterococcaceae*. This node is perfectly resolved (100/100, *ufboot/alrt* supports). A color palette is associated with *Enterococcus* genera in the GTDB. The trees were drawn with *iTOL* (Letunic and Bork, 2021).

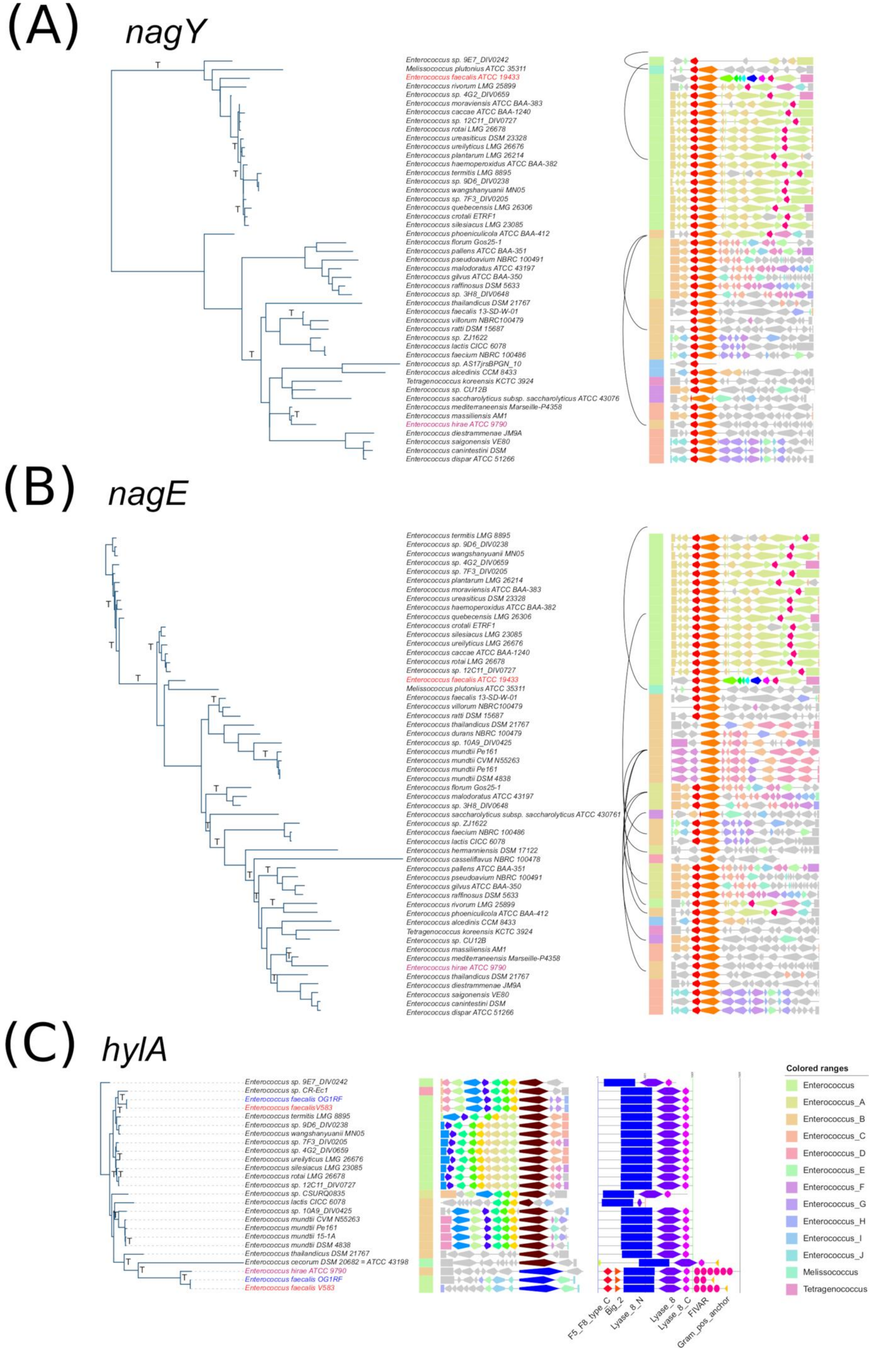
(A) nagY
Enterococcus sp. 9E7_DIV0242
Melissococcus plutonius ATCC 35311
Enterococcus faecalis ATCC 19433
Enterococcus rivorum LMG 25899
Enterococcus sp. 4G2_DIV0659
Enterococcus moraviensis ATCC BAA-383
Enterococcus caccae ATCC BAA-1240
Enterococcus sp. 12C11_DIV0727
Enterococcus rotai LMG 26678
Enterococcus ureasiticus DSM 23328
Enterococcus ureilyticus LMG 26676
Enterococcus plantarum LMG 26214
Enterococcus haemoperoxidus ATCC BAA-382
Enterococcus termitis LMG 8895
Enterococcus sp. 9D6_DIV0238
Enterococcus wangshanyuanii MN05
Enterococcus sp. 7F3_DIV0205
Enterococcus quebecensis LMG 26306
Enterococcus crotali ETRF1
Enterococcus silesiacus LMG 23085
Enterococcus phoeniculicola ATCC BAA-412
Enterococcus florum Gos25-1
Enterococcus pallens ATCC BAA-351
Enterococcus pseudoavium NBRC 100491
Enterococcus malodoratus ATCC 43197
Enterococcus gilvus ATCC BAA-350
Enterococcus raffinosus DSM 5633
Enterococcus sp. 3H8_DIV0648
Enterococcus thailandicus DSM 21767
Enterococcus faecalis 13-SD-W-01
Enterococcus villorum NBRC100479
Enterococcus ratti DSM 15687
Enterococcus sp. ZJ1622
Enterococcus lactis CICC 6078
Enterococcus faecium NBRC 100486
Enterococcus sp. AS17jrsBPGN_10
Enterococcus alcedinis CCM 8433
Tetragenococcus koreensis KCTC 3924
Enterococcus sp. CU12B
Enterococcus saccharolyticus subsp. saccharolyticus ATCC 43076
Enterococcus mediterraneensis Marseille-P4358
Enterococcus massiliensis AM1
Enterococcus hirae ATCC 9790
Enterococcus diestrammenae JM9A
Enterococcus saigonensis VE80
Enterococcus canintestini DSM
Enterococcus dispar ATCC 51266
(B) nagE
Enterococcus termitis LMG 8895
Enterococcus sp. 9D6_DIV0238
Enterococcus wangshanyuanii MN05
Enterococcus sp. 4G2_DIV0659
Enterococcus sp. 7F3_DIV0205
Enterococcus plantarum LMG 26214
Enterococcus moraviensis ATCC BAA-383
Enterococcus ureasiticus DSM 23328
Enterococcus haemoperoxidus ATCC BAA-382
Enterococcus quebecensis LMG 26306
Enterococcus crotali ETRF1
Enterococcus silesiacus LMG 23085
Enterococcus ureilyticus LMG 26676
Enterococcus caccae ATCC BAA-1240
Enterococcus rotai LMG 26678
Enterococcus sp. 12C11_DIV0727
Enterococcus faecalis ATCC 19433
Melissococcus plutonius ATCC 35311
Enterococcus faecalis 13-SD-W-01
Enterococcus villorum NBRC100479
Enterococcus ratti DSM 15687
Enterococcus thailandicus DSM 21767
Enterococcus durans NBRC 100479
Enterococcus sp. 10A9_DIV0425
Enterococcus mundtii Pe161
Enterococcus mundtii CVM N55263
Enterococcus mundtii Pe161
Enterococcus mundtii DSM 4838
Enterococcus florum Gos25-1
Enterococcus malodoratus ATCC 43197
Enterococcus sp. 3H8_DIV0648
Enterococcus saccharolyticus subsp. saccharolyticus ATCC 430761
Enterococcus sp. ZJ1622
Enterococcus faecium NBRC 100486
Enterococcus lactis CICC 6078
Enterococcus hermanniensis DSM 17122
Enterococcus casseliflavus NBRC 100478
Enterococcus pallens ATCC BAA-351
Enterococcus pseudoavium NBRC 100491
Enterococcus gilvus ATCC BAA-350
Enterococcus raffinosus DSM 5633
Enterococcus rivorum LMG 25899
Enterococcus phoeniculicola ATCC BAA-412
Enterococcus alcedinis CCM 8433
Tetragenococcus koreensis KCTC 3924
Enterococcus sp. CU12B
Enterococcus massiliensis AM1
Enterococcus mediterraneensis Marseille-P4358
Enterococcus hirae ATCC 9790
Enterococcus thailandicus DSM 21767
Enterococcus diestrammenae JM9A
Enterococcus saigonensis VE80
Enterococcus canintestini DSM
Enterococcus dispar ATCC 51266
(C) hylA
Enterococcus sp. 9E7_DIV0242
Enterococcus sp. CR-Ec1
Enterococcus faecalis OG1RF
Enterococcus faecalisV583
Enterococcus termitis LMG 8895
Enterococcus sp. 9D6_DIV0238
Enterococcus wangshanyuanii MN05
Enterococcus sp. 7F3_DIV0205
Enterococcus sp. 4G2_DIV0659
Enterococcus ureilyticus LMG 26676
Enterococcus silesiacus LMG 23085
Enterococcus rotai LMG 26678
Enterococcus sp. 12C11_DIV0727
Enterococcus sp. CSURQ0835
Enterococcus lactis CICC 6078
Enterococcus sp. 10A9_DIV0425
Enterococcus mundtii CVM N55263
Enterococcus mundtii Pe161
Enterococcus mundtii 15-1A
Enterococcus mundtii DSM 4838
Enterococcus thailandicus DSM 21767
Enterococcus cecorum DSM 20682 = ATCC 43198
Enterococcus hirae ATCC 9790
Enterococcus faecalis OG1RF
Enterococcus faecalis V583
F5_F8_type_C
Big_2
Lyase_8_N
Lyase_8
Lyase_8_C
FIVAR
Gram_pos_anchor
Colored ranges
Enterococcus
Enterococcus_A
Enterococcus_B
Enterococcus_C
Enterococcus_D
Enterococcus_E
Enterococcus_F
Enterococcus_G
Enterococcus_H
Enterococcus_I
Enterococcus_J
Melissococcus
Tetragenococcus

Supplementary Figure 2. Family trees for *nagY*, *nagE* and *hylA* with gene neighborhood. The three panels correspond to the **(A)** *nagY* orthologs, **(B)** *nagE* orthologs and **(C)** *hylA* homologs. Reconciled protein family trees were inferred with *GeneRax*, with the species tree and family alignment as input. The method takes into account the amino acids substitution model, duplications, losses and gene transfers. Predicted gene transfers are reported on the branches (T). The first column describes the taxonomy of the species corresponding to the proteins on the tree using the same color code as in Figure 3. The braces indicate inter-genera transfers. The chromosomal neighborhood of the genes is displayed alongside with a color code referring to the OGs of each gene that highlights the conservation. For the *hylA* homologs, the Pfam domain organization of the encoded proteins is shown.

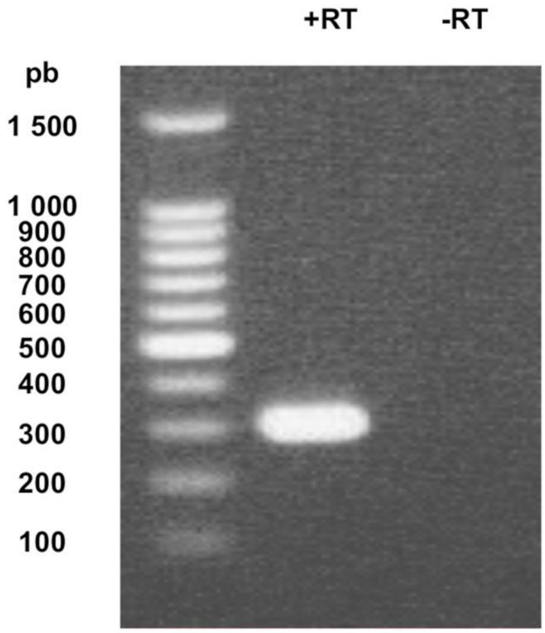

Supplementary Figure 3. Co-transcription of *nagY-nagE* operon evidenced by RT-PCR. RNAs were extracted from *E. faecalis* V19 strain in exponential culture phase, cultivated in cdM17 with 0.5% glucose. +/-RT: with or without addition of reverse transcriptase. Size of DNA ladder is indicated.

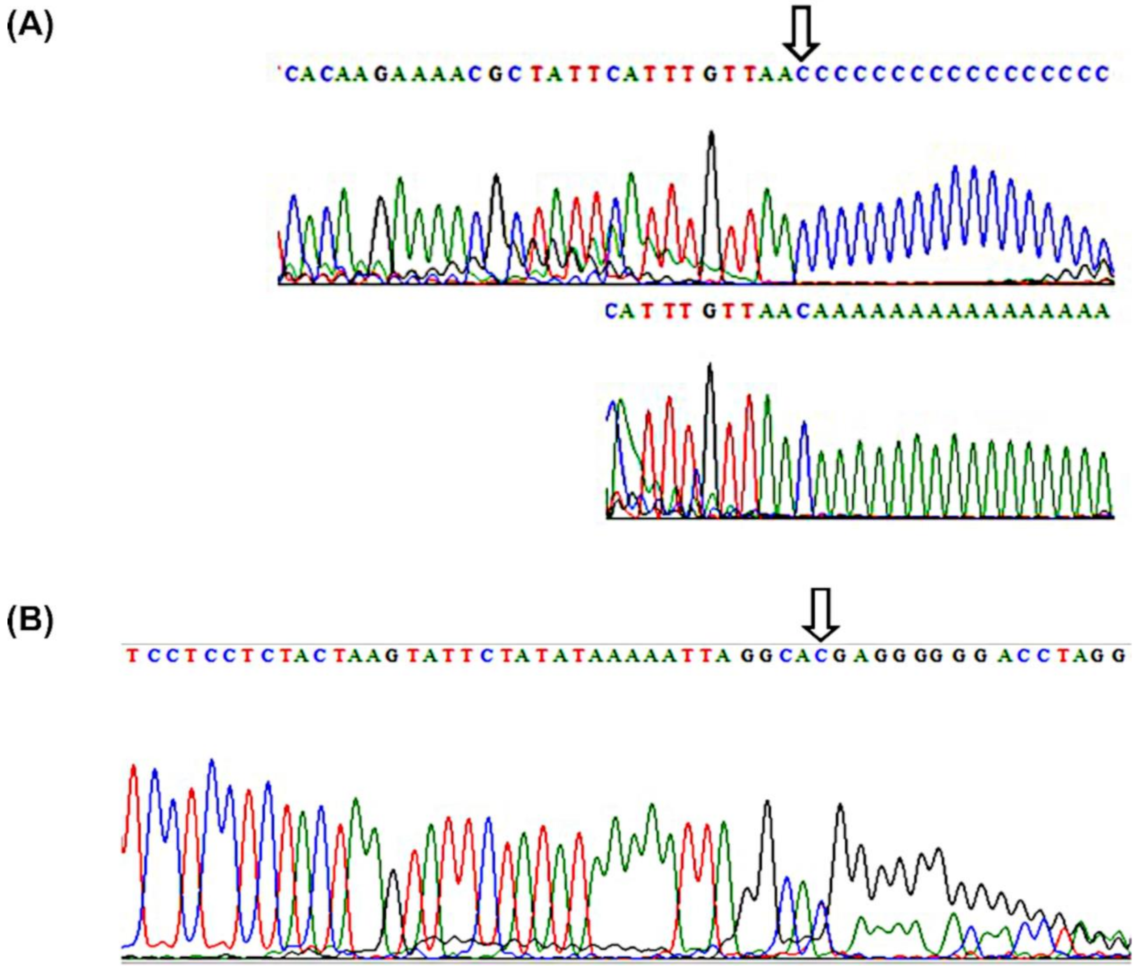

Supplementary Figure 4. Identification of the *nagY* and *hylA* transcription start site by 5'RACE-PCR assay. **(A)** *nagY-nagE* +1 of transcription was identified with a polyC and a polyA during RACE-PCR because of ambiguities on the C base **(B)** *hylA* +1 of transcription was identified with polyG. Transcriptional start sites are designed by arrows.

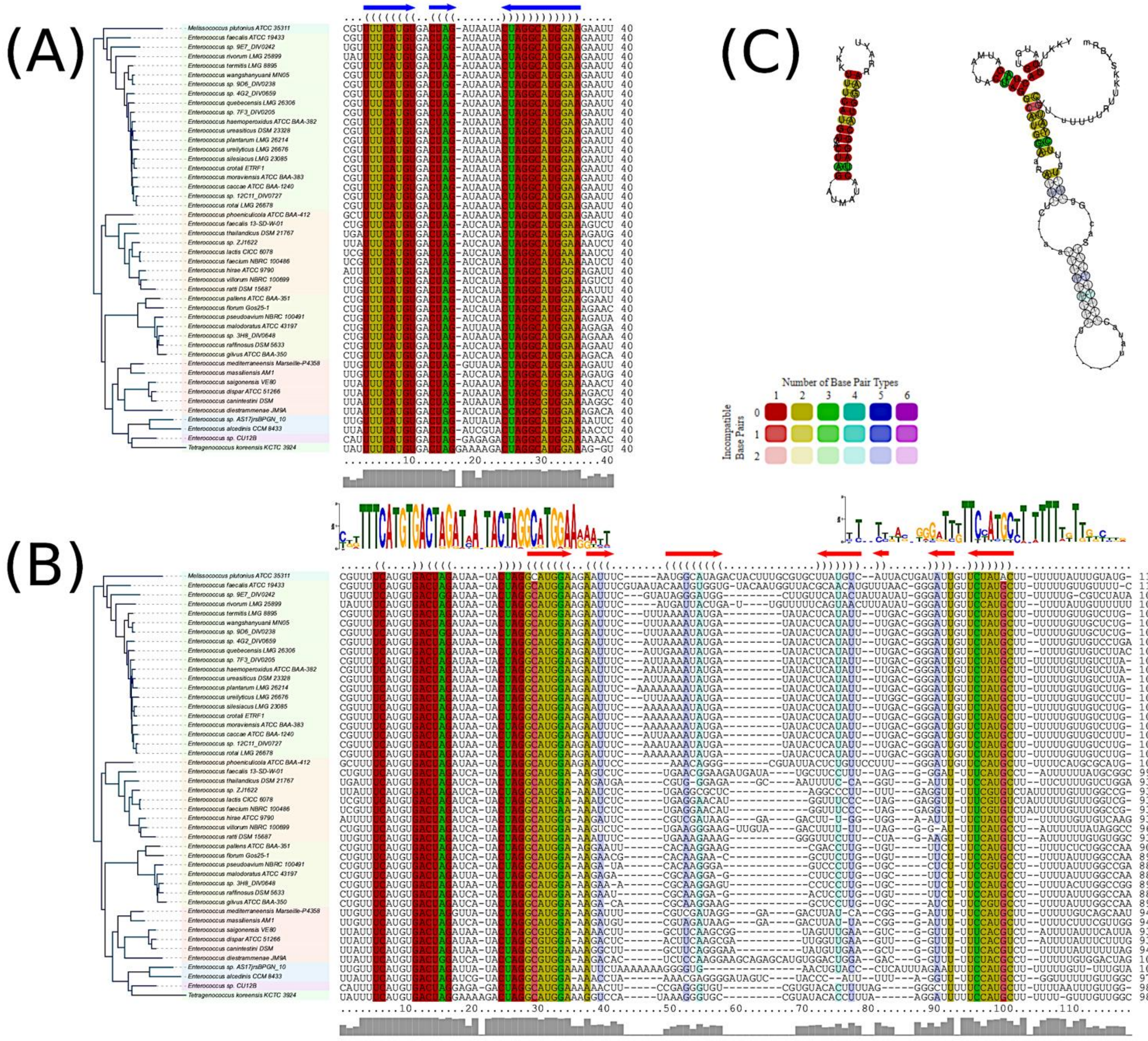

Supplementary Figure 5. Alignments and secondary structure of the RAT motifs and the rho-independent transcription terminator in enterococci. Sequences are ordered according to the species tree (Figure 3). Base pairs are indicated by parentheses. Consensus secondary structures are marked with arrows. In the alignments, colors indicate compatible base pairs. The shade shows the number of different types of C-G, G-C, A-U, U-A, G-U or U-G compatible base pairs in the corresponding columns. The hue shows the conservation of the base pair sequence. The saturation decreases with the number of incompatible base pairs. This coding reflects the structural conservation of the base pair. **(A)** Predicted alignment and structure for the RAT region. **(B)** Predicted alignment and structure for the rho-independent transcription terminator. The sequence logo from *MEME* motif 1 and 2 were placed above the alignment. Spaces (-) have been inserted into the logo to match the alignment. **(C)** Consensus secondary structures of region in A on the left, and in B on the right.

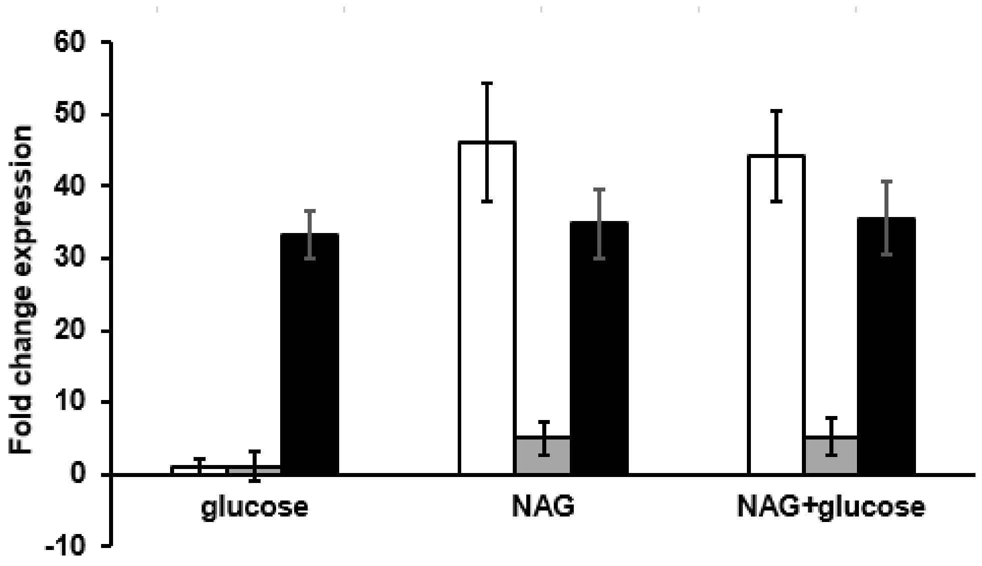

Supplementary Figure 6. Study of *nagE* induction of expression in presence of NAG or/and glucose. The *nagE* gene expression in WT (white), Δ*nagY* (grey), and Δ*5'nagY* (black) strains were revealed by RT-qPCR, with RNA purified from culture in presence of NAG or/and glucose as sole carbon source. The results were normalized with the expression of the housekeeping gene *gyrA* (gyrase). Error bars represent the standard error of triplicate measurements.

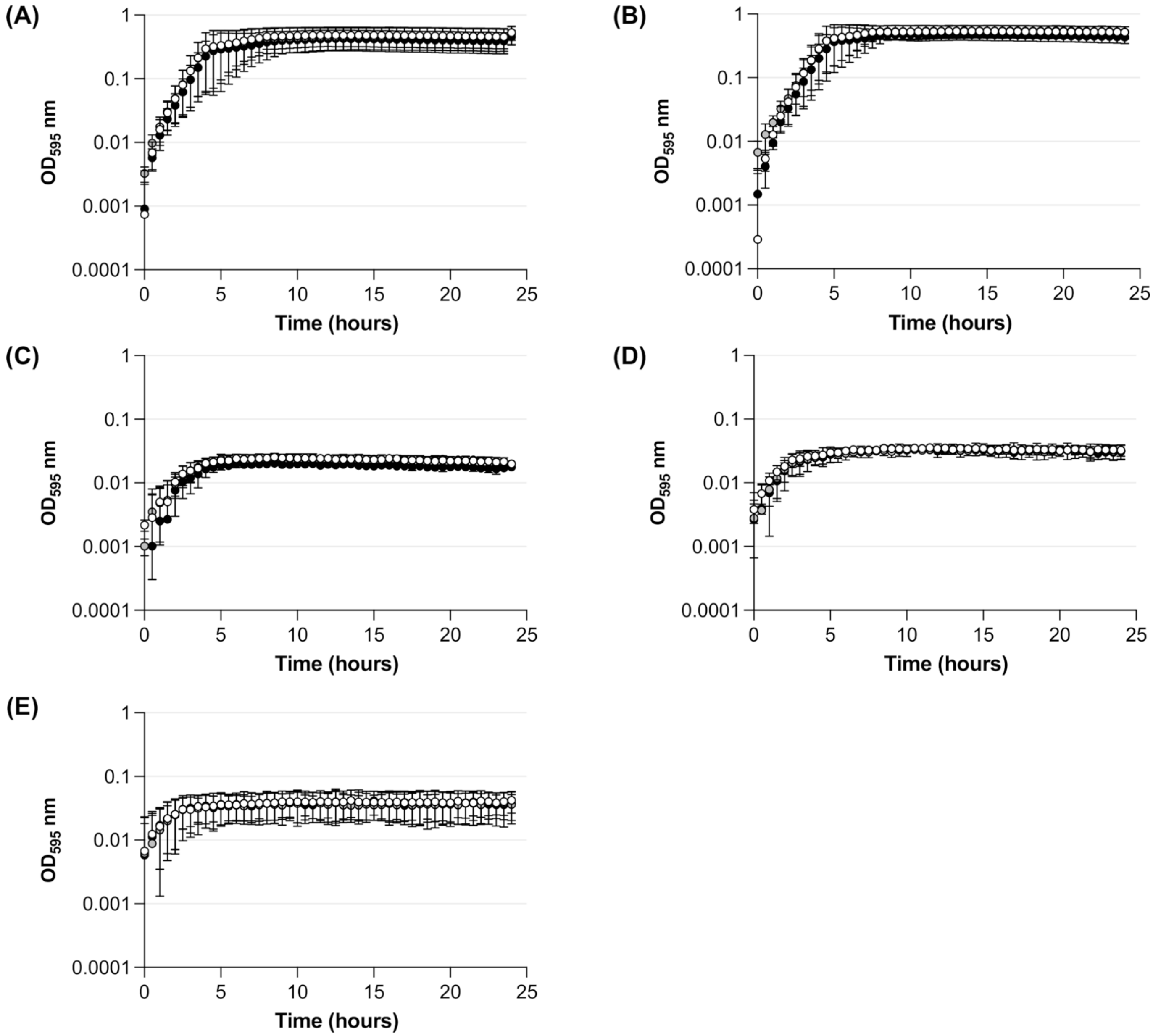

Supplementary Figure 7. Growth experiments in WT (white), *ΔnagY* (grey) and *ΔhylA* (black) strains with 0.5% **(A)** glucose, **(B)** NAG, **(C)** hyaluronic acid, **(D)** chondroitin sulfate or **(E)** heparin sodium. Error bars represent the standard error of at least 2 measurements.

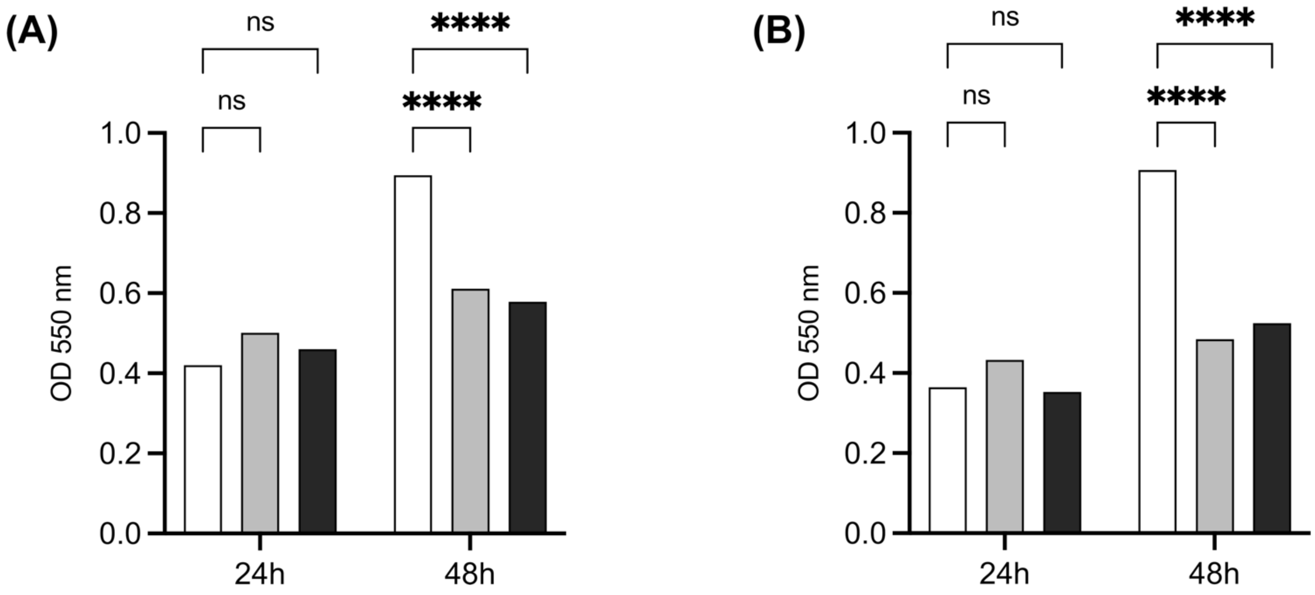

Supplementary Figure 8. Biofilm formation in WT (white), Δ*nagY* (grey) and Δ*hylA* (black) strains with the corresponding observation of crystal violet staining. *p*-value <0.0001 (Tukey's multiple comparisons test). A coating with 1 µg/mL of chondroitin sulfate **(A)** or heparin sodium **(B)** was performed before biofilm formation. The experiments were realized three times.

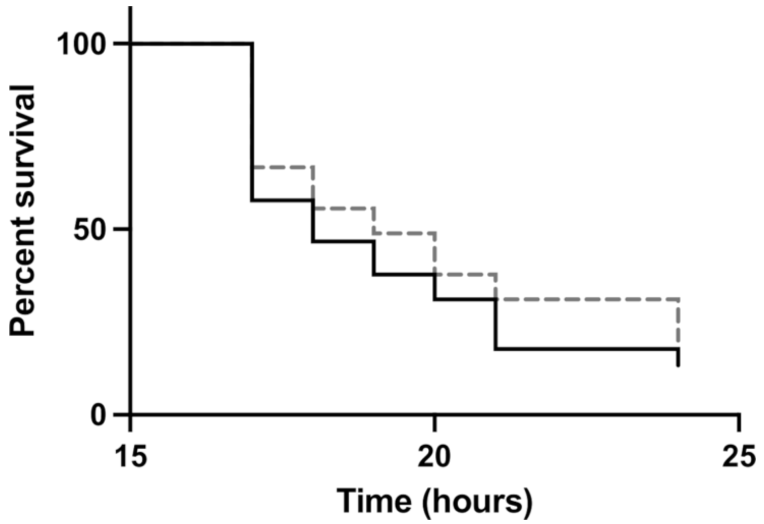

Supplementary Figure 9. Survival of *G. mellonella* larvae infected by *E. faecalis* WT (solid line) and Δ*nagY* (dashed grey line) strains. The experiment was performed three times using 15 caterpillars per strain and per test. No significative difference was observed (log-rank test).

References

Andrews, S. (2010). FastQC. Available at: https://www.bioinformatics.babraham.ac.uk/projects/fastqc/.

Guindon, S., Dufayard, J.-F., Lefort, V., Anisimova, M., Hordijk, W., and Gascuel, O. (2010). New algorithms and methods to estimate maximum-likelihood phylogenies: assessing the performance of *PhyML* 3.0. *Syst Biol* 59, 307–321. doi: 10.1093/sysbio/syq010.

Hoang, D. T., Chernomor, O., von Haeseler, A., Minh, B. Q., and Vinh, L. S. (2018). *UFBoot2:* improving the ultrafast bootstrap approximation. *Mol Biol Evol* 35, 518–522. doi: 10.1093/molbev/msx281.

La Carbona, S., Sauvageot, N., Giard, J.-C., Benachour, A., Posteraro, B., Auffray, Y., et al. (2007). Comparative study of the physiological roles of three peroxidases (NADH peroxidase, Alkyl hydroperoxide reductase and Thiol peroxidase) in oxidative stress response, survival inside macrophages and virulence of *Enterococcus faecalis*. *Mol. Microbiol.* 66, 1148–1163. doi: 10.1111/j.1365-2958.2007.05987.x.

Lebreton, F., Manson, A. L., Saavedra, J. T., Straub, T. J., Earl, A. M., and Gilmore, M. S. (2017). Tracing the enterococci from Paleozoic origins to the hospital. *Cell* 169, 849-861.e13. doi: 10.1016/j.cell.2017.04.027.

Letunic, I., and Bork, P. (2021). Interactive Tree Of Life (*iTOL*) v5: an online tool for phylogenetic tree display and annotation. *Nucleic Acids Res* 49, W293–W296. doi: 10.1093/nar/gkab301.

Minh, B. Q., Schmidt, H. A., Chernomor, O., Schrempf, D., Woodhams, M. D., von Haeseler, A., et al. (2020). *IQ-TREE 2*: new models and efficient methods for phylogenetic inference in the genomic era. *Mol Biol Evol* 37, 1530–1534. doi: 10.1093/molbev/msaa015.

Parks, D. H., Rinke, C., Chuvochina, M., Chaumeil, P.-A., Woodcroft, B. J., Evans, P. N., et al. (2017). Recovery of nearly 8,000 metagenome-assembled genomes substantially expands the tree of life. *Nat Microbiol* 2, 1533–1542. doi: 10.1038/s41564-017-0012-7.

Paulsen, I. T., Banerjei, L., Myers, G. S. A., Nelson, K. E., Seshadri, R., Read, T. D., et al. (2003). Role of mobile DNA in the evolution of vancomycin-resistant *Enterococcus faecalis*. *Science* 299, 2071–2074. doi: 10.1126/science.1080613.

Thurlow, L. R., Thomas, V. C., and Hancock, L. E. (2009). Capsular polysaccharide production in *Enterococcus faecalis* and contribution of CpsF to capsule serospecificity. *J Bacteriol* 191, 6203–6210. doi: 10.1128/JB.00592-09.

Tonkin-Hill, G., MacAlasdair, N., Ruis, C., Weimann, A., Horesh, G., Lees, J. A., et al. (2020). Producing polished prokaryotic pangenomes with the *Panaroo* pipeline. *Genome Biol* 21, 180. doi: 10.1186/s13059-020-02090-4.

Zhou, Z., Charlesworth, J., and Achtman, M. (2020). Accurate reconstruction of bacterial pan- and core genomes with *PEPPAN*. *Genome Res* 30, 1667–1679. doi: 10.1101/gr.260828.120.
